# Native American geography shaped historical fire frequency in forests of eighteenth-century Pennsylvania, USA

**DOI:** 10.1038/s41598-023-44692-5

**Published:** 2023-10-30

**Authors:** Stephen J. Tulowiecki, Brice B. Hanberry, Marc D. Abrams

**Affiliations:** 1 1 College Circle, SUNY Geneseo, Geneseo, NY 14423 USA; 2 USDA Forest Service, Rocky Mountain Research Station, Rapid City, SD 57702 USA; 3 204 Forest Resources Bldg., Department of Ecosystem Science and Management, The Pennsylvania State University, University Park, PA 16802 USA

**Keywords:** Biogeography, Fire ecology, Forest ecology

## Abstract

Researchers have debated the relative importance of environmental versus Indigenous effects on past fire regimes in eastern North America. Tree-ring fire-scar records (FSRs) provide local-resolution physical evidence of past fire, but few studies have spatially correlated fire frequency from FSRs with environmental and anthropogenic variables. No study has compared FSR locations to Native American settlement features in the eastern United States. We assess whether FSRs in the eastern US are located near regions of past Native American settlement. We also assess relationships between distance to Native American settlement, environmental conditions, and fire frequency in central Pennsylvania (PA), US, using an “ensemble of small models” approach for low sample sizes. Regression models of fire frequency at 21 locations in central PA often selected distance-based proxies of Indigenous land use. Models with mean annual temperature and Native American variables as predictors explained > 70% of the variation in fire frequency. Alongside temperature and wind speed, “distance to nearest trail” and “mean distance to nearest town” were significant and important predictors. In 18th-century central PA, fires were more frequent near Indigenous trails and towns, and further south due to increasing temperature and pyrophilic vegetation. However, for the entire eastern US, FSRs are located far from past settlement, limiting their effectiveness in detecting fire patterns near population centers. Improving understanding of historical fire will require developing FSRs closer to past Native American settlement.

Much research has examined the relative importance of climate versus disturbance, including anthropogenic burning, upon pre-Euro-American forests of eastern North America^1–5^. Some believe that climate has largely driven fire and vegetation dynamics^2^ via lightning frequency, fuel moisture, fuel loads, and the oscillation between moist periods of fuel accumulation and dry periods conducive to fire. However, others posit that Indigenous-caused disturbance, especially cultural burning, has maintained vegetation in a pyroclimax state for millennia^4,6^. Researchers have implicated fire exclusion in the decline of fire-tolerant xerophytic vegetation^4,7^, but many climate-related and other causes have been proposed^8,9^.

A related debate concerns the geographic extent of Indigenous land use, including fire, in modifying past forests in eastern North America^1,6,10,11^. Native Americans historically utilized various strategies to boost land productivity, such as in-place promotion of perennial plant species, creation of habitats for game, and transplantation of favored plant species^12–14^. They ignited low-intensity fires in forests, woodlands, and grasslands for purposes such as procuring food and easing travel^15^. Varying methodologies suggest that burning and related vegetation modifications occurred along travel corridors and within 10–50 km of towns^16–19^.

Tree-ring fire-scar records (FSRs) have allowed researchers to establish fire chronologies, estimate fire return intervals, determine fire seasonality, and create correlative spatial models of fire frequency^6,20–22^. Fire scars result from non-lethal injuries to tree trunks that kill cambial cells that are covered by subsequent growth; scars form in moderate-severity fires or along the outskirts of high-severity fires^22^. Findings from fire-scar chronologies suggest cultural burning in eastern North America with fire return intervals as low as 3–6 years^6^. These authors reported that most fire scars in the eastern US are produced during the dormant season when lightning is uncommon, suggesting anthropogenic ignition. Fire intervals from FSRs may be minimum-frequency estimates where frequent low-severity fires occur, because such fires may injure few to no trees, and because scars are rare from consecutive annual burns due to reduced fuels in the second year^23^. While FSRs may not capture small fires, studies have confirmed their reliability for reconstructing landscape-scale fire histories through modern comparisons between FSRs and other sources such as fire perimeter maps^24^.

Previous researchers have used correlative modeling to disentangle climatic and anthropogenic influences upon past geographic patterns of fire-adapted plants and ecosystems in the eastern US, but have rarely spatially modeled fire frequency directly. Using correlative spatial models, distance-based proxies of Indigenous land use (e.g., distance to nearest town or trail) have improved models of past pyrophilic vegetation^18,25–27^. Elsewhere, work has used correlative modeling (e.g., regression) to model mean fire interval but without Native American land-use proxies^28^. Stambaugh et al.^20^ used regression to model historical fire return interval from FSRs in Missouri versus terrain roughness, human population density, and distance to river travel corridors, representing a rare effort toward quantitatively testing relationships between fire frequency or mean fire interval obtained from FSRs and Native American settlement. Elsewhere, Guyette et al.^21^ developed a model of US fire frequency from FSRs based on oxygen availability, temperature, and precipitation, but did not include anthropogenic predictors of fire.

Geographic comparisons between Native American settlement and FSR-based fire frequency can assess where burning occurred, such as whether burning occurred close to population centers and travel routes, and whether selective patch burning^29^ or broadcast burning over large geographic extents was common. Another framework proposes that burning occurred only close to Native American towns and within barrens driven by geology or other microsite characteristics^30^. Others have proposed the “yard and corridor” concept whereby burning occurred in patches connected by travel corridors also maintained by fire^31,32^. Comparing FSR-based fire frequency estimates to Indigenous settlement in eastern North America would also help quantify the geographic extent of cultural burning while also testing the relative effect of climate and terrain conditions upon fire frequency.

In this study, we first examine the geographic correspondence between FSRs and Native American settlement to assess how well FSRs may capture past Indigenous burning. This purpose covers temperate forests and northern mixed woods of the eastern US and southeastern Canada^33,34^. Second, we assess whether distance-based proxies of Native American land use improve understanding of historical fire frequency via correlative spatial models, and study the effect of proximity to Native American settlement on FSR-based fire frequency. We use models to infer the relative importance of environmental factors versus Native American land use on fire frequency. The study region for the second purpose is central Pennsylvania. We pursue 18th- and early 19th-century case studies because cultural burning during this time is expected to be recorded in FSRs, and because research has mapped features of settlement for this period.

## Methods

### Comparing FSR locations to Native American settlement

We quantified distance between FSRs and contemporaneous 18th- and early 19th-century Native American settlement in eastern North America. We made comparisons with (1) towns in the eastern US for circa 1760–1790; (2) towns in the Great Lakes region circa 1810; and (3) towns, trails, and canoe-navigable waterways in 18th-century PA. We chose comparisons based on the availability and reliability of settlement data; no unified Native American settlement dataset exists for eastern North America. We chose some sources^35,36^ after examining other map atlases on Native American history. For all comparisons described below, we also qualitatively compared town locations to current estimated forest establishment dates (i.e., age of representative overstory trees)^37^ to assess which locations with older forests close to towns could be targeted in future FSR development. Many sources of towns and trails described below were maps that required georeferencing and tracing to convert into GIS-format layers. For tasks involving geographic information systems (GIS) software we used ArcGIS Pro^38^.

We downloaded FSR locations^39^ and selected FSRs with earliest rings that predated settlement features mapped and that were situated within the geographic extent of the settlement data source. For the first two comparisons, we also chose FSRs less affected by early Euro-American settlement by excluding those in counties with a mean population density ≥0.8 persons km^2^ (≥2 persons mi^2^) at the end of a period (i.e., 1790 or 1820; see comparisons one and two below) according to US Census-based population estimates^40^; the US Census defined “unsettled” areas as those with densities below this threshold^41^.

For comparison one, we mapped towns circa 1760–1790 CE (*n* = 377) covering the eastern US using maps compiled from primary traveler accounts, scholarly works, French and English military correspondence, Smithsonian Bureau of American Ethnology reports, Indian Claims Commission documents, and historical society publications^35^. The maps showed only principal towns in some regions and exhaustive town locations elsewhere. As inferred from town names, in some cases town locations represented movement of the same community over time. Positional error relative to the extent of analysis was believed to be low (mean approximately <5 km). For comparison two, we mapped agricultural and fishing towns circa 1810 CE (*n* = 318) using a map of the Great Lakes region compiled from primary accounts, reports, documents, and historical publications from Great Lakes states^36^. We did not pursue mapping exact locations but assumed positional error was similarly low. Comparison three involved comparisons between FSRs and 18th-century towns, trails, and canoe-navigable waterways in PA (see next section for data descriptions).

### Modeling historical fire frequency in central Pennsylvania

To test the effect of proximity to Native American settlement on fire frequency recorded in FSRs, we developed models of fire frequency based on climate, terrain, and Native American variables for central Pennsylvania (PA). Data preparation was performed with ArcGIS Pro^38^, whereas modeling was performed using R^42,43^.

#### Central Pennsylvania study area

Pennsylvania has the highest number of FSRs (*n* = 31) of any state completely within our study area and has scholarly resources on Native American town and trail locations. Eighteenth-century PA was a period of declining Native American population due to epidemics and conflict^44^, with likely implications for subsistence economies and fire regimes. Nevertheless, thousands to tens of thousands of Native Americans occupied the present-day area of PA, including the Delaware, Iroquois, and Shawnee, at over 130 towns^45^. Towns were focused along major river valleys, such as the Susquehanna, Delaware, and Allegheny Rivers. This analysis focused on an approximately 24,000-km^2^ portion of central PA defined by a rectangle around FSR locations, covering portions of the Ridge-and-Valley and Allegheny Plateau physiographic provinces, and possessing a humid continental climate that circa 1900 ranged in mean annual temperature from 5.5 to 10.9 °C and in mean annual precipitation from 82 to 122 cm^46^. Pre-Euro-American forests were predominantly composed of maple (*Acer* spp.), American beech (*Fagus grandifolia*), and eastern hemlock (*Tsuga canadensis*) in northern forests of the Allegheny Plateau; and of oak (*Quercus* spp.), pine (*Pinus* spp.), hickory (*Carya* spp.), and American chestnut (*Castanea dentata*) in eastern temperate forests of the Ridge-and-Valley in the south^47,48^.

#### Data collection: fire frequency

We determined fire frequency 1701–1750 for FSRs in central PA (*n* = 21) using fire-scar history diagrams in published studies^44,49–51^. We counted years with ≥ 1 scar, in years with ≥2 trees; published diagrams were legible and often labeled years with ≥ 1 scar. Scars formed predominantly during the dormant season. We did not find other published literature or datasets with FSR data 1701–1750 for central PA beyond those associated with the North American Tree-Ring Fire-Scar Network^22,39^. We chose this period because it preceded Euro-American settlement at all FSR locations^44^, and to mitigate the effects of recording wartime fire use during the Beaver Wars and French and Indian War. We did not model mean fire interval because applying existing rules to some FSRs (e.g., including a fire interval if more than half of it spanned the period of interest) yielded zero fire intervals within the 1701–1750 period despite having fires. About 14% of intervals that would have been included according to these rules included years outside of the 1701–1750 period, affecting 43% of FSR sites; 24% of sites had zero fire intervals entirely within this period.

#### Data collection: predictors of fire frequency

We considered 32 Native American, bioclimatic, and terrain predictors of fire frequency. After consulting literature for predictors of fire frequency, and addressing collinearity by removing predictors that were correlated with others at Pearson’s *r* values ≥ |0.70|^52^, we kept 12 predictors (Table 1) to test whether fires were more frequent near Native American settlement, in warmer/drier climates, in windier locations, in gentler terrain, and at lower elevations with higher O_2_ concentrations.

**Table 1:** Predictors for regression models of fire frequency in central Pennsylvania 1701–1750

Category	Variable	Unit	Description	Spatial resolution	Base source(s)
Climate	Mean annual temperature	°C	Mean annual temperature from 1895 to 1924	4 km	^46^
Climate	Mean annual precipitation	mm	Mean annual precipitation from 1895 to 1924	4 km	^46^
Climate	Mean wind speed	m s^-1^	Mean wind speed at 10 m height from 2008 to 2017	250 m	^102^
Native American	Mean distance to nearest town	km	Mean distance to nearest Native American town between 1701 and 1750	100 m	Various; see text
Native American	Distance to nearest trail	km	Distance to nearest 18th-century Native American trail	100 m	^61^
Native American	Distance to nearest fifth-order stream	km	Distance to nearest estimated canoe-navigable waterway	100 m	^63^
Native American	Distance to nearest sixth-order stream	km	Distance to nearest estimated canoe-navigable waterway	100 m	^63^
Topographic	Terrain ruggedness index (TRI)	m	Mean of the absolute differences in elevation between a cell and its 8 neighboring cells	90 m	^64^
Topographic	Vector ruggedness measure (VRM)	(unitless)	Captures variability of both terrain slope and terrain aspect	90 m	^64^
Topographic	Mean TRI	m	Mean TRI (see above) within a 3-km radius	90 m	^64^
Topographic	Mean VRM	(unitless)	Mean VRM (see above) within a 3-km radius	90 m	^64^
Topographic	Elevation	m	Elevation above sea level	30 m	^103^

For descriptive statistics of these predictors, see Table 2.

We created a “mean distance to Native American towns 1701–1750” predictor. We first digitized towns in PA^45^, portions of New York^53–55^, and Maryland^56,57^. We also recorded approximate years of town settlement. The PA source^45^ was compiled from over 20 sources including major works on PA Indigenous history. We then calculated distance to nearest town for each year 1701–1750 as raster-format layers, and averaged these layers together. Based on comparisons with locations of towns in archaeological site files^58^, we believe positional error of towns and trails (see below) was low (mean approximately <3 km). We did not augment the dataset with additional town sites from state historic preservation office files, because many town locations in those files were already mapped in the PA source used^45^, or lacked temporal data regarding dates of occupation. We focused on towns and travel routes versus other settlement features (e.g., camps, cemeteries) because previous local-scale studies have detected relationships between fire-tolerant vegetation and town or trail locations^18,25,27,59^.

Other variables captured proximity to travel routes. We calculated the distance between FSRs and nearest circa 18th-century trail in GIS software. We digitized trails from maps by Wallace^60,61^, a source for mapping trails utilized in past FSR-based studies^62^. Wallace^60,61^ synthesized archival sources from the PA Bureau of Land Records and the Division of Land Records at Harrisburg to map trails, and refined trail locations by consulting topographic maps and through fieldwork. His sources included early maps, journals of early Euro-American captives and explorers, written and oral histories, archaeological evidence, and early survey and land office documents. Wallace^61^ stated that error “was not often very high” in mapping trail locations. We digitized locations of estimated canoe-navigable waters by investigating stream order data from the National Hydrography Dataset^63^; modern canoe-navigable waters generally correspond to fifth-order streams and higher.

For mean annual precipitation and temperature, we calculated means for 1895–1924^46^ to capture relative climatic patterns preceding 20th-century global warming. Whereas terrain ruggedness index (TRI) and vector ruggedness measure (VRM) were provided^64^, we calculated means within a three-kilometer radius to quantify terrain ruggedness in a neighborhood similar to previous work^20^. We excluded soil moisture because all FSR sites were located on well-drained soils and because fire-maintained vegetation can occur on soils of varying moisture levels in the eastern US^65^. We also considered including relative abundance of pre-Euro-American pyrophilic trees^48^ as a predictor, but we excluded it because it was collinear with temperature and because pyrophilic vegetation is both an effect and cause of fire^66^.

We also included two measures of sampling bias: number of trees sampled, and area sampled (in hectares). Doing so explored the possibility that characteristics of FSR sites affected the number of fires recorded, either that more trees sampled or a larger area sampled would produce more fire scars and higher fire frequency. Previous research has shown relationships between fire frequency, mean fire interval, and area sampled^67^.

#### Regression model development

We developed univariate and bivariate regression models; previous research has used regression to model pre-Euro-American fire frequency^20,28^. We trained linear and Poisson model sets with each single predictor, and every unique combination of two predictors. In each set, we kept univariate models when the predictor possessed a *p*-value < 0.05, and bivariate models when both predictors possessed a *p*-value < 0.10. Given issues concerning spatially-clustered FSRs, and differences in geographic area and number of trees sampled, we used various schemes for weighting training data observations, including unweighted, weighting by number of trees per FSR site, weighting by area sampled at an FSR site, and down-weighting spatially-clustered sites (i.e., by giving a cluster of ten sites the same total weight as other sites). We examined common regression outputs such as Akaike Information Criterion (AIC), *R*^2^, coefficients, and coefficient *p*-values. For Poisson models we calculated deviance-based *R*^2^.

To visualize spatial predictions of fire frequency, we adopted an “ensemble of small models” approach from the species distribution modeling literature^68^. This paradigm overcomes limitations of small sample sizes by developing univariate and bivariate models that adhere to the “10:1 rule” (i.e., ten or more observations for every one predictor). A prediction is calculated as a weighted average of individual model predictions based upon a model performance metric. For each set (i.e., linear and Poisson), we weighted each model’s predictions by its *R*^2^. For model predictions involving distance to nearest trail, we estimated trail locations^60,61^ in GIS software and calculated distance as a gridded raster layer. To assess overall performance for each ensemble, we calculated mean absolute deviation (MAD) and root-mean-square error (RMSE) between actual number of fires recorded in FSRs and ensemble model predictions, and Pearson’s *r* between actual and predicted fires. We also mapped model residuals for the linear and Poisson ensemble models.

## Results

### FSR locations versus Native American settlement

**Figure 1:**
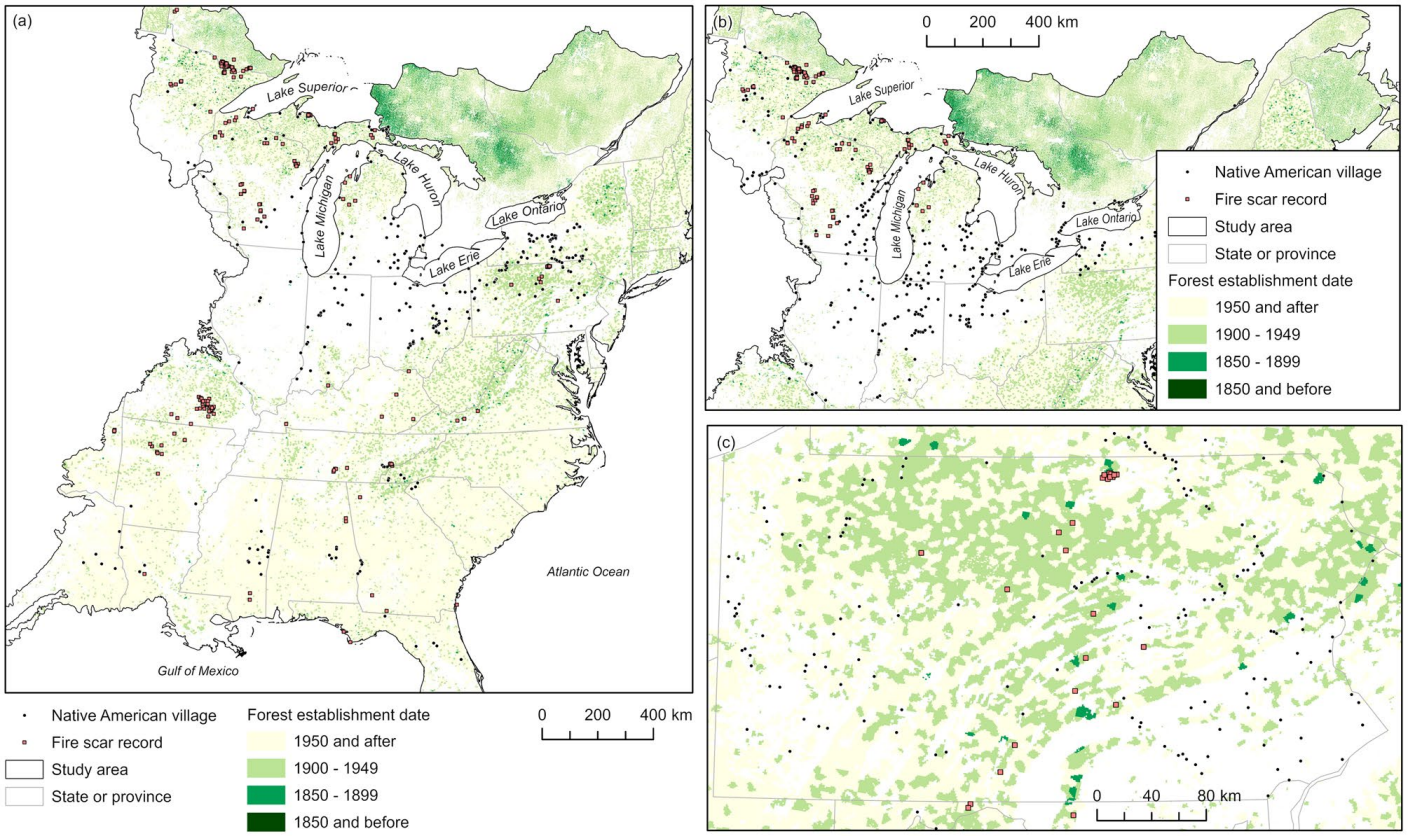
Native American towns in (**a**) eastern North America 1760–1790^35^, (**b**) the Great Lakes region 1810^36^, and (**c**) Pennsylvania (PA) 18th century (see text for sources), along with contemporaneous tree-ring fire-scar record (FSR) sites (see text for selection criteria). See Fig. 4–5 for an excerpt of 18th-century trails in central PA. Only towns and FSR sites within eastern North America are shown. Also shown is estimated forest age^37^.

FSRs were distant from Native American settlement (i.e., towns and town clusters), but the landscape-extent comparison revealed that FSRs were close to travel corridors. In the eastern US 1760–1790 (Fig. 1a), FSRs (*n* = 244) were a median of 116 km from the nearest town, with a range of 9–489 km (IQR = 50–146 km). Approximately 25% of FSRs were found within 50 km of towns, and just 7% were within 20 km. The northern Great Lakes (e.g., Chippewa territory), southern Appalachians (e.g., Cherokee), and central PA (e.g., Seneca Iroquois) had the greatest geographic correspondence between towns and FSRs 1760–1790. Conversely, the southern Great Lakes (e.g., Wyandot, Potawatomi, Ottawa, Moravian, Delaware, Shawnee) contained numerous towns but virtually no FSRs. The following regions possessed many towns, few FSRs, and older and more continuous forests, thereby having greater potential for developing new FSRs: portions of central New York, northwestern PA, southern Ohio, and the southern Appalachians.

In the Great Lakes region 1800–1820 (Fig. 1b), there was a closer spatial correspondence between FSRs and towns but the two were still distant. FSRs (*n* = 168) were a median of 32 km from the nearest town, with a range of 1–119 km (IQR = 27–46 km). Approximately 78% of FSRs were found within 50 km of towns, and 17% were within 20 km. The Great Lakes 1800–1820 exhibited similar characteristics as the previous comparison: towns and FSRs were more proximate in the northern Great Lakes (e.g., Menominee, Winnebago, Ojibwa, Ottawa) region but FSRs were virtually nonexistent in the southern Great Lakes (e.g., Miami, Wyandot, Potawatomi, Delaware) region. The following regions possessed many towns, few FSRs, and older and more continuous forests: portions of the Midwestern states, southwestern New York, and northwestern PA.

The landscape-scale comparison from central PA (Fig. 1c) revealed that while FSRs were generally far from Native American towns, they were closer to travel corridors. FSRs recording 1701–1750 (*n* = 21) were a median of 25 km from the nearest town, with a range of 8–42 km (IQR = 23–27 km). All FSRs were within 50 km of nearest town, but just 14% were within 20 km. Whereas those numbers described distance to nearest 18th-century town, other results described mean distance to nearest town. The median of mean distances between FSRs and towns 1701–1750 was 60 km, with a range of 32–96 km (IQR = 56–71 km). Northwestern PA again emerged as an area with Native American settlement and older forests today, but without FSRs. On the whole, FSRs were much closer to travel corridors, being a median of 3 km from trails (range = 1–10 km, IQR = 1–4 km), 3 km from nearest fifth-order stream (range = 1–11 km, IQR = 2–4 km), and 13 km from nearest sixth-order stream (range = 2–27 km, IQR = 5–15 km).

### Ensemble models of Central PA fire frequency

Distance-based proxies of Native American land use were important predictors of fire frequency 1701–1750 in central PA. Univariate and bivariate models implied that fire was more frequent closer to trails and towns, in warmer and windier locations, and in less complex terrain. Models trained using different observation weighting schemes produced generally similar results (see Supplementary Tables S1 and S2). Table 2 summarizes fire frequency and other predictors at FSR locations and within a rectangle around FSR locations, and summarizes conditions for eastern temperate versus northern forests. Based on FSRs, fire frequency 1701–1750 ranged from 0–14 fires (interquartile range [IQR] = 2–5 fires) equaling 0–2.8 fires per decade (IQR = 0.4–1.0 fires per decade), with a median of 3 fires (0.6 fires per decade; *n* = 21).

**Table 2:** Descriptive statistics of central Pennsylvania (PA) based on (a) ≈1000 equally-spaced points within a rectangle around fire-scar record (FSR) locations and (b) conditions at FSR sites (*n* = 21)

Predictor or condition	Mean			Median			Minimum			Maximum			25th percentile			75th percentile		
	ALL	ETF	NF	ALL	ETF	NF	ALL	ETF	NF	ALL	ETF	NF	ALL	ETF	NF	ALL	ETF	NF
(a) Based on ≈1000 equally-spaced points																		
Distance to nearest town, 18th century (km)	22.5	20.6	27.3	21.3	19.7	27.6	0.7	0.7	1.3	54.0	54.0	50.7	13.3	11.6	18.7	30.9	27.9	36.3
Mean distance to nearest town, 1701–1750 (km)	63.6	59.7	73.0	66.3	60.9	72.0	16.5	16.5	42.4	102.1	102.1	101.2	52.3	46.4	67.0	76.0	74.2	79.3
Distance to nearest trail, 18th century (km)	4.2	3.5	6.0	3.1	2.4	5.1	0.0	0.0	0.0	20.2	16.6	20.2	1.3	1.1	2.6	6.0	5.0	8.7
Distance to nearest fifth-order stream (km)	5.7	5.8	5.4	4.7	4.7	4.7	0.0	0.0	0.0	24.0	24.0	17.6	2.1	1.9	2.4	8.3	8.7	7.9
Distance to nearest sixth-order stream (km)	14.0	13.4	15.4	11.6	11.0	13.4	0.0	0.0	0.0	46.0	46.0	45.5	4.9	4.5	5.8	21.5	21.1	23.8
Mean annual temperature, 1895–1924 (°C)	8.5	9.1	7.0	8.5	9.5	6.9	5.5	5.5	5.7	10.9	10.9	8.7	7.1	8.4	6.5	9.7	10.0	7.5
Mean annual precipitation, 1895–1924 (mm)	970	959	997	965	946	1003	823	825	823	1217	1217	1140	924	916	972	1008	989	1030
TRI (m)	11	9	15	8	7	14	0	0	2	56	35	56	4	4	7	15	12	22
VRM	0.003	0.002	0.006	0.001	0.001	0.003	0.000	0.000	0.000	0.039	0.022	0.039	0.000	0.000	0.001	0.004	0.002	0.008
Mean TRI (m)	11	9	15	10	9	15	1	1	5	27	23	27	8	7	12	14	12	18
Mean VRM	0.003	0.002	0.005	0.002	0.002	0.005	0.000	0.000	0.001	0.013	0.008	0.013	0.001	0.001	0.004	0.004	0.003	0.007
Elevation (m)	379	317	530	365	281	545	91	91	178	775	775	769	223	202	458	522	425	613
Mean wind speed (m s^-1^)	3.1	3.1	3.2	3.0	3.0	3.1	0.8	1.3	0.8	7.6	7.6	6.3	2.4	2.4	2.3	3.6	3.5	3.8
Pyrophilic trees, circa 18th century (% of total)	68%	81%	36%	85%	88%	24%	2%	3%	2%	97%	97%	90%	56%	82%	6%	90%	91%	66%
(b) Based on FSR site locations																		
Distance to nearest town, 18th century (km)	24.7	22.7	25.8	25.3	22.5	26.3	7.6	7.6	17.5	42.4	42.4	32.7	22.5	19.0	25.0	27.4	24.7	27.4
Mean distance to nearest town, 1701–1750 (km)	61.3	59.3	62.6	60.1	54.9	60.2	32.0	32.0	56.2	95.7	95.7	75.3	56.2	48.5	57.7	71.3	73.1	62.2
Distance to nearest trail, 18th century (km)	3.5	4.1	3.0	3.1	2.5	3.2	0.5	0.9	0.5	10.3	10.3	5.7	1.4	1.0	2.1	4.2	6.9	3.9
Distance to nearest fifth-order stream (km)	3.6	4.6	3.0	2.6	2.9	2.6	0.6	1.2	0.6	11.3	11.3	5.8	1.7	1.8	1.7	3.7	5.6	3.7
Distance to nearest sixth-order stream (km)	11.4	10.2	12.1	12.8	10.3	13.9	1.9	1.9	3.6	27.0	27.0	16.4	5.5	4.1	11.9	14.9	12.1	15.2
Mean annual temperature, 1895–1924 (°C)	7.9	9.2	7.1	7.3	9.6	7.1	6.6	7.5	6.6	10.2	10.2	7.3	7.1	9.0	6.8	9.3	9.8	7.2
Mean annual precipitation, 1895–1924 (mm)	940	996	905	913	978	886	827	913	827	1135	1135	1068	862	936	862	1023	1037	890
TRI (m)	19	15	22	19	14	23	4	4	6	34	28	34	12	9	15	25	19	32
VRM	0.008	0.007	0.009	0.007	0.005	0.008	0.001	0.001	0.002	0.023	0.019	0.023	0.005	0.003	0.006	0.009	0.009	0.009
Mean TRI (m)	15	12	16	15	12	16	10	11	10	20	14	20	13	11	15	16	13	16
Mean VRM	0.004	0.003	0.005	0.004	0.003	0.004	0.002	0.002	0.004	0.009	0.005	0.009	0.004	0.002	0.004	0.005	0.004	0.005
Elevation (m)	554	510	581	555	480	585	402	402	521	649	649	637	521	439	536	625	578	625
Mean wind speed (m s^-1^)	3.8	4.1	3.6	3.7	4.6	3.3	1.2	1.2	1.2	6.8	6.8	6.7	2.5	2.5	2.5	5.4	5.5	4.2
Pyrophilic trees, circa 18th century (% of total)	51%	88%	29%	35%	91%	19%	8%	73%	8%	96%	96%	80%	19%	87%	19%	90%	92%	35%
Number of fires, 1701–1750	4.0	6.6	2.4	3.0	6.0	2.0	0.0	1.0	0.0	14.0	14.0	5.0	2.0	3.5	1.0	5.0	9.0	4.0

All conditions described in this table are predictors in regression models except for number of fires (the dependent variable), distance to nearest town (18th century), and percent pyrophilic trees (pre-Euro-American). For full predictor descriptions, see Table 1. ALL = entire central PA, ETF = eastern temperate forests, NF = northern forests.

Across the four model weighting schemes, nine predictors appeared at least once in chosen linear models (Supplementary Table S1); in order from most to least chosen they are: distance to nearest trail (negatively correlated with fire frequency; increasing distance led to decreased fire frequency), mean annual temperature (positively correlated), mean wind speed (positive), mean VRM (negative), mean distance to nearest town (negative), VRM (positive), mean TRI (typically negative), elevation (negative), and TRI (negative). Three predictors did not appear in any linear models given our selection criteria: mean annual precipitation, distance to nearest 5th-order stream, and distance to nearest 6th-order stream. More Poisson models were selected and exhibited a higher number of significant relationships with predictors than linear models (Table S2); all predictors appeared at least once in Poisson models. The following predictors were the top five most chosen in Poisson models: distance to nearest trail (negatively correlated with fire frequency), mean wind speed (positive), mean VRM (typically negative), mean distance to nearest town (negative), and TRI (negative). Mean annual temperature and distance to nearest trail formed seven of the top models within each of the eight unique combinations of model type (i.e., linear or Poisson) and weighting scheme. Across all models, 4 linear models and 13 Poisson models yielded an *R*^2^ ≥ 0.70, of which 3 linear models and 2 Poisson models included a Native American variable; all models with *R*^2^ ≥ 0.70 included temperature.

**Figure 2:**
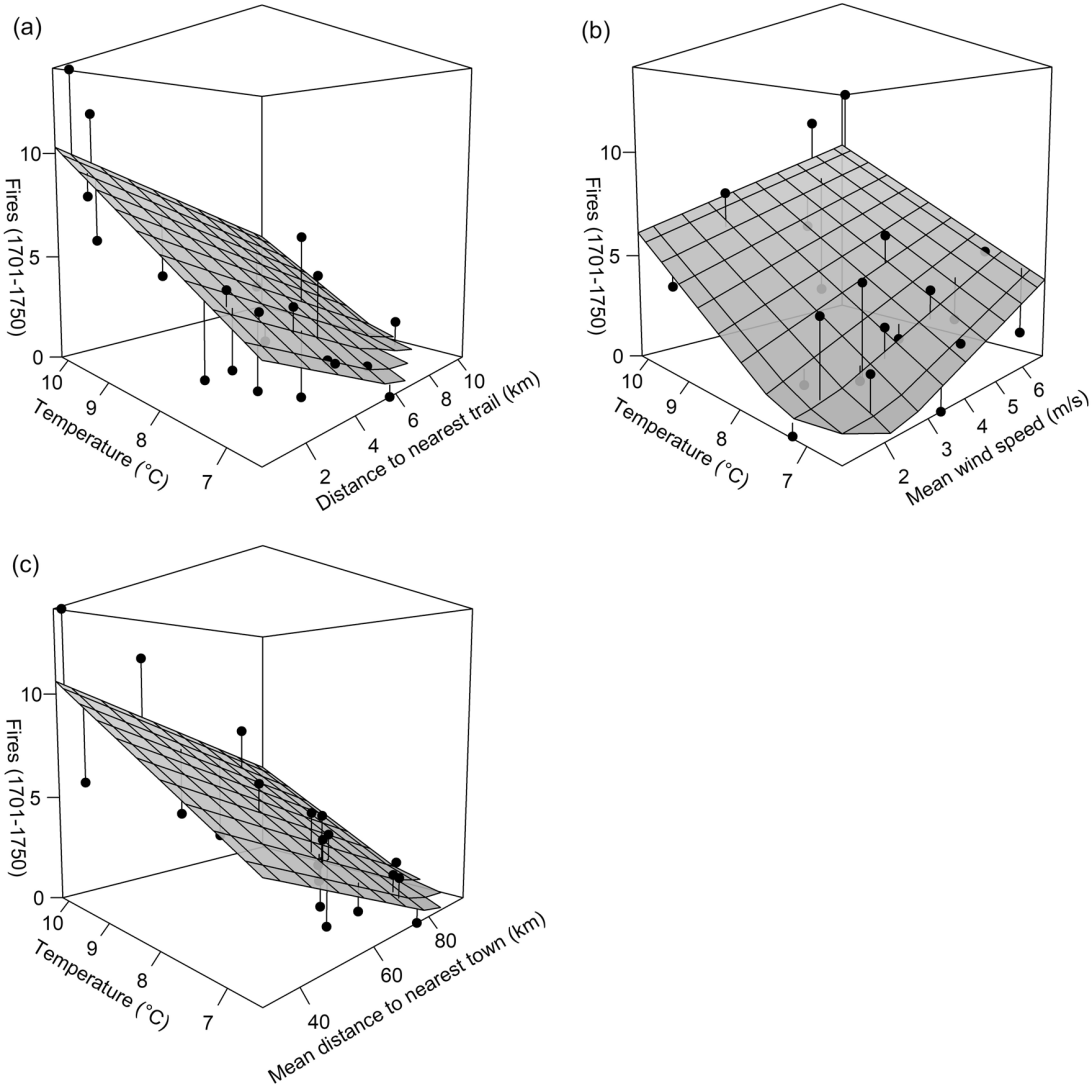
Three-dimensional scatterplots and regression trend surfaces for the top three linear regression models (with spatially-weighted observations).

**Figure 3:**
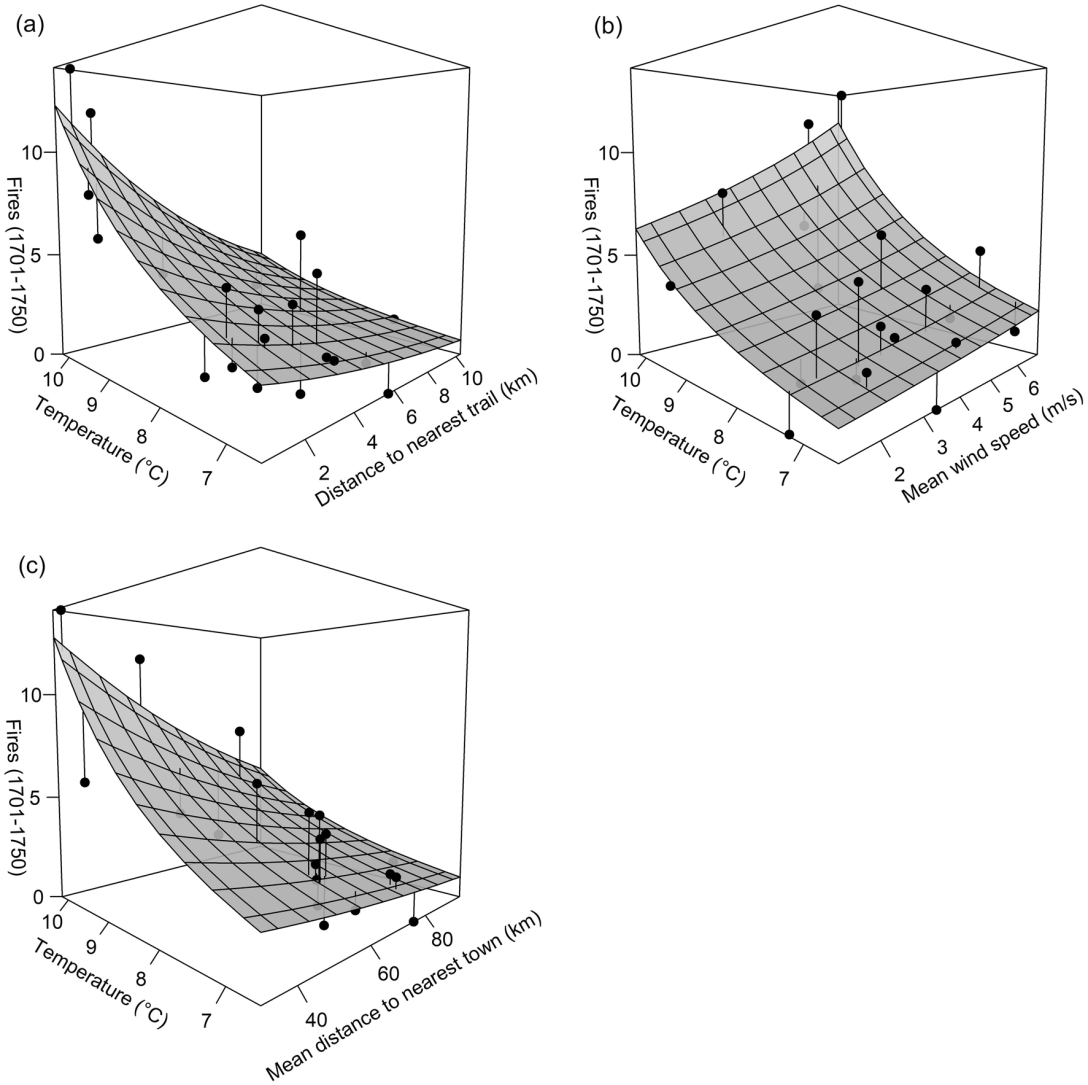
Three-dimensional scatterplots and regression trend surfaces for the top three Poisson regression models (with spatially-weighted observations).

The weighting scheme in which the ten spatially-clustered FSRs in the northern portion of central PA were down-weighted (see Fig. 1c later) produced the most retained models given our criteria; those results are presented throughout the remainder of this section to provide a more detailed examination of models. Mean annual temperature was consistently positively correlated with fire frequency in all models that included it (Figs. 2 and 3; Tables 3 and 4). It was generally the most important variable overall, appearing the second-most often of any predictor in the top ten linear models (Table 3) and most often in the top ten Poisson models (Table 4). Temperature also produced the top univariate model in each set (linear model *R*^2^ = 0.567; Poisson model *R*^2^ = 0.616). Percent pyrophilic trees was excluded as a predictor from all model sets because it was highly collinear with temperature (*r* = 0.81). Areas of higher fire frequency corresponded with warmer temperatures and higher percentages of pyrophilic trees in central PA: at FSRs in eastern temperate forests, median values were 9.6 °C, 91% pyrophilic trees, and 6.0 fires (1.2 fires per decade); but in northern forests, median values were 7.1 °C, 19% pyrophilic trees, and 2.0 fires (0.4 fires per decade).

**Table 3:**
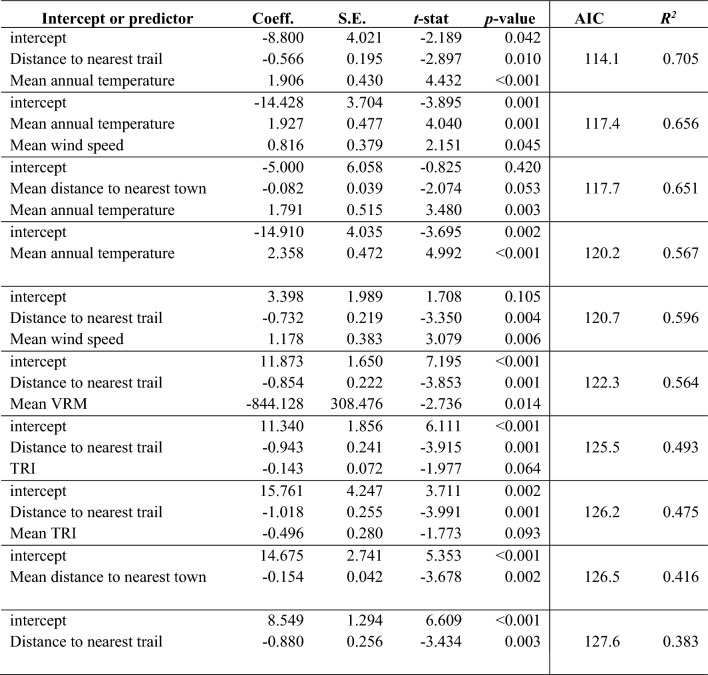
Top ten linear regression models (with spatially-weighted observations) of fire frequency in central Pennsylvania 1701–1750.

For all linear models, see Supplementary Table S1.

**Table 4:**
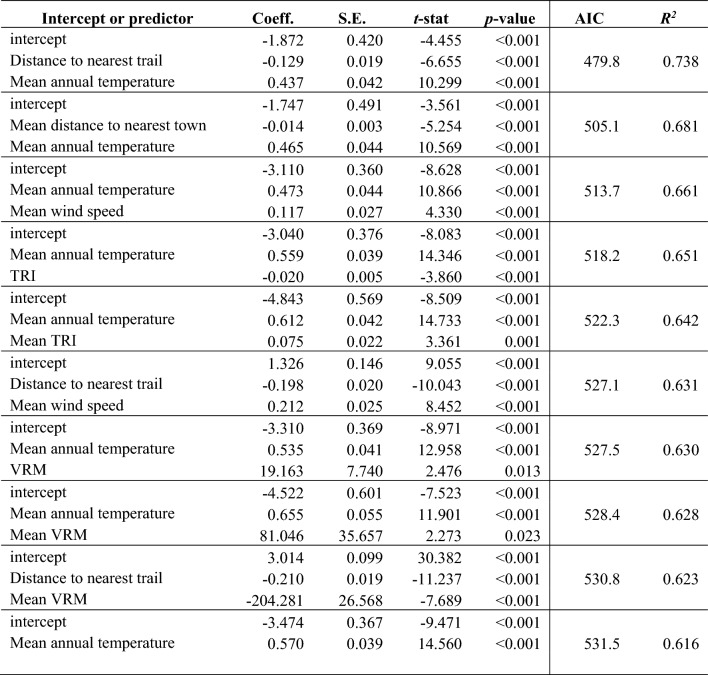
Top ten Poisson regression models (with spatially-weighted observations) of fire frequency in central Pennsylvania 1701–1750

*R*^*2*^ = deviance-based *R*^*2*^. For all Poisson models, see Supplementary Table S2.

**Figure 4:**
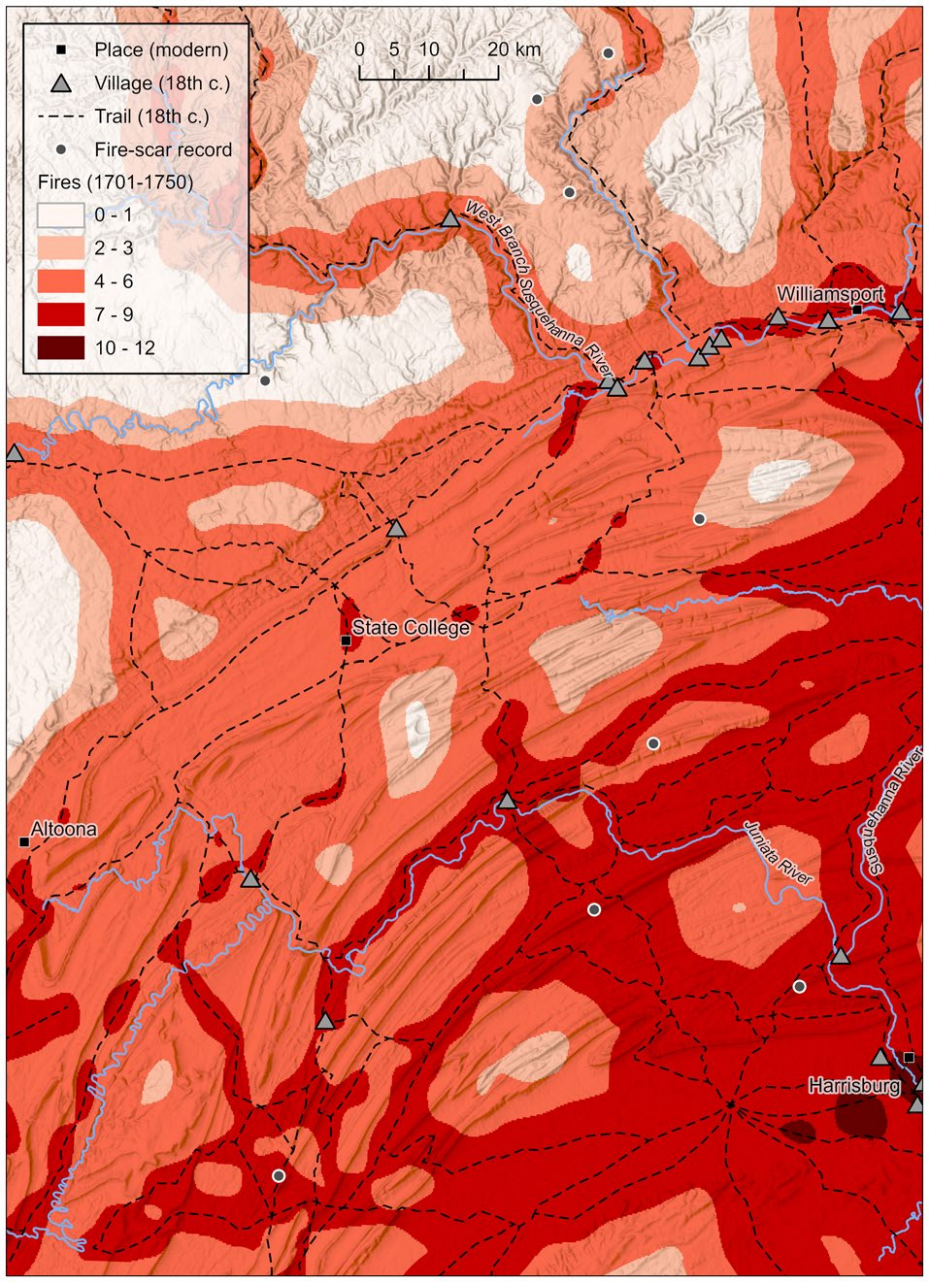
Ensemble linear regression model prediction of fire frequency (i.e., number of fires 1701–1750) in central Pennsylvania (based on models with spatially-weighted observations). Ensemble model predictions of <0 fires were reassigned a value of 0 fires.

**Figure 5:**
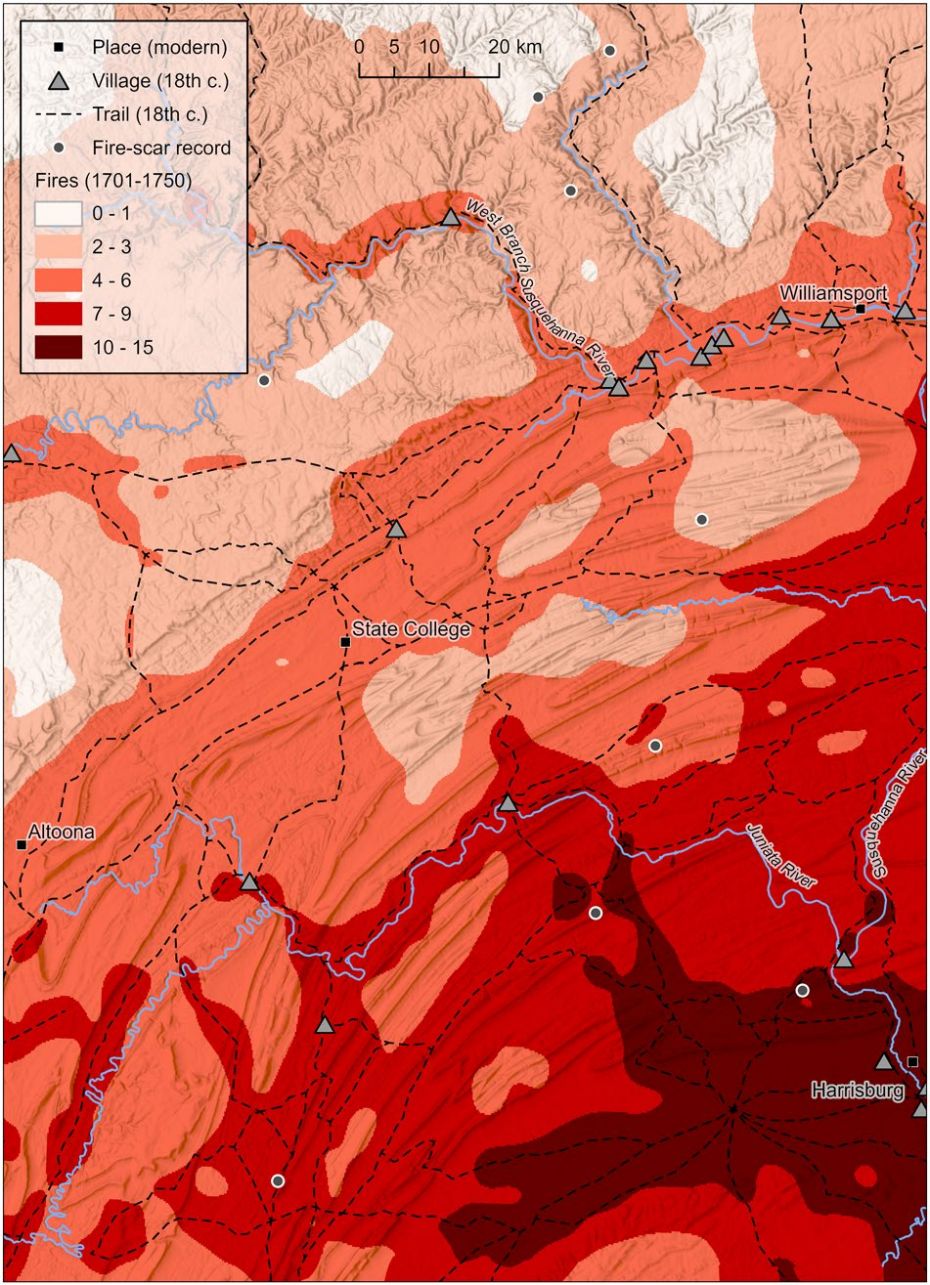
Ensemble Poisson regression model prediction of fire frequency (i.e., number of fires 1701–1750) in central Pennsylvania (based on models with spatially-weighted observations).

Proxies of Native American land use were consistently negatively correlated with fire frequency: fire was more frequent closer to trails and towns (Figs. 2, 3, 4 and 5; Tables 3 and 4). Distance to nearest trail was generally the second-most important predictor, appearing most often of any predictor in the top ten linear models and second-most often in the top ten Poisson models (i.e., with spatially-weighted observations). Temperature and distance to nearest trail were predictors in the best linear (*R*^2^ = 0.705) and Poisson (*R*^2^ = 0.738) models. Mean distance to nearest town was slightly less important, being present in two out of the top ten linear models and one of the top ten Poisson models. Top *R*^2^ values for models with mean distance to nearest town (and with temperature) as a predictor were *R*^2^ = 0.651 for linear models and *R*^2^ = 0.681 for Poisson models.

Mean wind speed was consistently positively correlated with fire frequency in all linear and Poisson models to include the predictor (Tables 3 and 4). It was roughly the third-most important predictor, present in two of the top ten linear models and two of the top ten Poisson models (i.e., with spatially-weighted observations). Top *R*^2^ values for models with wind speed (and with temperature) as a predictor were *R*^2^ = 0.656 for linear models and *R*^2^ = 0.661 for Poisson models.

Compared to predictors described above, terrain predictors were less important and/or did not always exhibit consistently positive or negative relationships with fire frequency. Appearing in the top ten linear models (i.e., with spatially-weighted observations) with *p* < 0.10 were mean VRM (negatively correlated within top ten models), mean TRI (negatively correlated), and TRI (negatively correlated); TRI and mean VRM were consistent in the directionality of their relationship with fire frequency across all linear models including those outside the top ten. TRI was consistently negatively correlated with fire frequency in Poisson models that included it. Mean VRM was most often negatively correlated with fire frequency across all Poisson models, but in the top-performing model to include this predictor (with temperature) it was positively correlated. Mean TRI was both positively and negatively correlated with fire frequency across Poisson models. VRM appeared in linear models outside of the top ten and was positively correlated; it appeared in several Poisson models and was also positively correlated.

Least important were elevation, mean annual precipitation, distance to nearest fifth-order stream, and distance to nearest sixth-order stream, which did not appear in any linear models (i.e., with spatially-weighted observations) given our selection criteria. Elevation (positively/negatively correlated), mean annual precipitation (negatively correlated), distance to nearest fifth-order stream (positively/negatively correlated), and distance to nearest sixth-order stream (positively correlated) appeared in Poisson models outside of the top ten.

The ensemble of the top ten linear models (i.e., with spatially-weighted observations) suggested elevated fire frequency in eastern temperate forests of the southern two-thirds of central PA, with modeled fire frequency values as high as 10.0 fires (2.0 fires per decade) during the 1701–1750 period with an IQR of 4.5–7.2 fires (0.9–1.4 fires per decade; Fig. 4). In northern forests, modeled frequency peaked at 5.7 fires (1.1 fires per decade) with an IQR of 1.1–3.6 fires (0.2–0.7 fires per decade). The ensemble revealed large expanses with zero fires in northern forests, except for areas along trails approximately 20 km wide with fire frequencies generally around 1–5 fires (0.2–1.0 fires per decade). The MAD between actual fires recorded in FSRs and ensemble linear model prediction was 2.0 fires, the RMSE was 2.4 fires, and the Pearson’s *r* value between actual and predicted number of fires was 0.77. Models not in the top ten possessed adjusted *R*^2^ < 0.35. Mapping the residuals of both the linear and Poisson (discussed next) model ensembles showed no consistent spatial patterns of systematic over- or under-prediction in fire frequency.

The ensemble of the top ten Poisson models (i.e., with spatially-weighted observation) showed similar spatiotemporal patterns in fire frequency as the linear model ensemble (Fig. 5). Modeled fire frequency values in eastern forests reached 14.8 fires (3.0 fires per decade) from 1701–1750 with an IQR of 3.9–8.0 fires (0.8–1.6 fires per decade). In northern forests, modeled frequency peaked at 4.3 fires (0.9 fires per decade) with an IQR of 1.4–2.5 fires (0.3–0.5 fires per decade). The Poisson model ensemble similarly revealed large expanses with few fires in northern forests, except for areas along trails. The ensemble Poisson model predictions fit the training data slightly better than ensemble linear model predictions: the MAD was 1.8 fires, the RMSE was 2.2 fires, and the Pearson’s *r* value between actual and predicted fires was 0.79. Models not in the top ten possessed *R*^2^ < 0.61.

Models including measures of sampling bias (i.e., number of trees sampled; area sampled) generally were poorer-fitting models, did not manifest significant relationships with bias measures, and/or were omitted given our model selection criteria. Similar to above, the following results pertain to linear models with spatially-weighted observations (see Methods), but similar results were also obtained with unweighted models. Out of linear models, the univariate model with number of trees sampled as a predictor was significant (*p* = 0.018; *R*^2^ = 0.261), whereas the univariate linear model with area sampled was non-significant (*p* = 0.563; *R*^2^ = 0.018). Only three bivariate linear models yielded near-significant (*p* < 0.10) relationships with number of trees, and these models still yielded significance with these predictors: mean distance to nearest town (*p* = 0.006; *R*^2^ = 0.523), distance to nearest trail (*p* = 0.014; *R*^2^ = 0.476), and mean wind speed (*p* = 0.006; *R*^2^ = 0.517). No bivariate linear models with area sampled as a predictor were kept, nor was area sampled ever significant or near-significant. Measures of sampling bias were more commonly significant in Poisson models, and models with these measures were more often retained given our selection criteria. Despite these results, number of trees was almost excluded as a variable due to collinearity with mean annual temperature (*r* = 0.59) and elevation (*r* = − 0.65).

Summary evidence also suggests the importance of distance to towns and trails, alongside environmental conditions, on fire frequency. The FSR with the most fires (i.e., 14 fires 1701–1750) had the second-lowest mean distance to nearest town (33 km), lowest distance to any 18th-century town (8 km), fourth-lowest distance to a trail (1 km), second-warmest mean annual temperature (10.1 °C), and was windiest (6.8 m s^-1^). Conversely, there were two FSRs with zero fires recorded in the 50-year period. These FSRs were farther on average from towns (61 and 75 km), farther from the nearest town (21 and 26 km), and farther from nearest trail (4 and 6 km). One FSR with zero fires exhibited the coolest mean annual temperature (6.6 °C), and the other possessed the second-lowest mean wind speed (1.2 m s^-1^).

## Discussion

We compared circa 18th- and early 19th-century Native American geography to FSRs for the eastern US, Great Lakes, and central PA. We then assessed whether climate, terrain, and distance-based Native American land-use proxies were correlated with historical fire frequency for central PA. This study produced two key findings. First, historical fire frequency was correlated with distance to Native American towns and trails along with temperature and other environmental predictors, suggesting that interlocking anthropogenic and environmental factors determined fire regimes. Second, FSRs were typically located far from major centers of Native American settlement at both regional and landscape scales but were close to trails.

In contrast to previous studies in the northeast US^2^, model results suggest the importance of Native Americans in fire application and maintaining fire-dependent vegetation. Wildland burning controlled tree densities, creating and maintaining ecosystems of grasslands and open forests^69,70^ that supported Native American subsistence economies and facilitated travel. Multiple lines of evidence are accumulating that Indigenous peoples managed many ecosystems of the eastern US with fire^6,13^, including results of this study.

### Climate-human-fire relationships and overall model performance

Though FSRs were generally distant from Native American settlement, this analysis revealed relationships between historical fire frequency, proxies of Native American land use, and environmental conditions in a region with a dense enough FSR network to infer such relationships (Figs. 2 and 3; Tables 3 and 4). Results support previous findings that fires or fire-maintained vegetation were more frequent near Native American settlement^20^, in warmer climates^21^, and in windier locations^65^. It offers some support for the idea that fire was more frequent in gentler terrain^20,65^. Results suggest that within the study area and surrounding region, both climate and humans were major determinants in fire frequency and distribution, with anthropogenic fire playing a larger role in warmer southerly climates^6^. Indigenous land use near towns and travel routes markedly enhanced fire frequency (Figs. 2, 3, 4 and 5) and therefore likely altered vegetation patterns. Studies finding no relationship between past vegetation and Indigenous land use based solely on proximity to archaeological site locations^19,71^ may be incorrect due to the omission of travel routes from analysis. Burning along travel corridors, and shifting corridors over centuries to millennia with changes in settlement patterns^72^, Indigenous peoples may have altered vegetation in most locations that were seasonally dry enough to ignite^16^.

Including proxies of Native American land use improved models of fire frequency (e.g., Tables 3 and 4). *R*^2^ values for models with these proxies exceeded those in regression models of fire frequency based on topographic roughness and human population density^20^, or based on environmental predictors only^28^. Proxies improved upon models that considered environmental variables only (Tables 3 and 4), just as similar predictors improved models of historical vegetation patterns in the eastern US^18,25–27^. Our ensemble predictions of historical fire frequency across central PA (Figs. 4 and 5) generally agree with previous predictions^21^ but predict higher frequency closer to past settlement.

Our models indicated elevated fire frequency near trails, and proximity to trails was generally more influential on fire frequency than proximity to towns in this study (Tables 3–4; Figs. 2, 3, 4 and 5). Firsthand historical accounts in the eastern US speculated on Indigenous use of fire along corridors to ease travel by thinning forests and maintaining grasslands^73^, and current traditional use of fire in a linear manner has been observed globally^74^. Correlation with distance to nearest trail supports previous research that found relationships between historical fire-tolerant vegetation and travel corridors within northern forests of northwestern PA^18^ and southwestern New York^25^, and supports research that linked burning captured in FSRs with travel corridors^17,31^. This study generally supports the “yard and corridor” concept^31,32^ for climates less favorable for fire (i.e., northern forests), whereby large burned areas near settlements are connected by travel corridors maintained by fire. For example, just northwest of the study area and prior to Euro-American settlement, a band of pyrophilic vegetation was found along the Allegheny River, a major travel corridor with late 18th-century towns^18,75^. The roughly 20 km-wide fire corridor predicted in northern forests along lower-elevation valleys (Figs. 2, 3, 4 and 5) is consistent with previous spatial estimates of Native American fire-maintained silvicultural patches^76^. Though fire corridors around trails may not be wide, results suggest that the dense network of travel routes facilitated extensive burning (Figs. 4 and 5). As for warmer eastern temperate forests, models suggested more widespread fire along trails and beyond warmer valleys due to enhancement by warmer temperatures.

Mean distance to nearest town was also important, supporting previous eastern US studies that emphasized relationships between vegetation and proximity to towns^18,25–27^. This study found a significant relationship at mean distances of roughly 30–100 km, suggesting that burning still occurred at far average distances from towns. Of Native American variables, only predictors recording distance to nearest canoe-navigable waterway were not significant, contradicting a previous study^20^. This result may have been due to inadequately mapping canoe-navigable routes, or because shallow inaccessible rivers and lack of traditional canoe-building materials discouraged water-based travel in 18th-century central PA^61^.

Though proxies of Native American land use were important, temperature was the most important predictor of fire frequency 1701–1750 in central PA (Figs. 2 and 3; Tables 3 and 4). Temperature influences the behavior, frequency, fuel structure, seasonality, and reaction rates of fire; the production and decay of woody fuels; and species composition^21^. Warm temperature prepares fuels such as downed coarse or fine woody debris: it lowers the amount of heat needed to raise fuel temperature to ignition, lowers relative humidity, and dries fuels^77^. However, the importance of temperature may also implicate pyrophilic vegetation as an additional driver of fire, because relative abundance of pyrophilic trees (Table 2) was correlated with temperature at FSRs in central PA. Associations between fire frequency, temperature, and Native American variables suggest a positive feedback that enhanced fire frequency in central PA. Warmer temperatures facilitated widespread cultural burning, and warmer temperatures and cultural burning also encouraged warmer-climate pyrophilic vegetation (e.g., *Quercus* spp.) that initiated a positive feedback with fire^66^ via fire-encouraging adaptations such as flammable leaf and needle litter^78^. Temperature may therefore measure climatic favorability for burning plus pyrophilic vegetation resulting from Indigenous burning, meaning that anthropogenic fire-mediated disturbance is even more important than suggested by the model ensembles. Moreover, warmer temperatures, in themselves, are not an ignition source for fire; those are provided by humans (frequently) and lightning (rarely) in much of the eastern US^13^.

Wind was the second-most important environmental predictor of fire frequency in models (Figs. 2 and 3; Tables 3 and 4). Wind provides oxygen to fire, determines fire direction along with terrain slope, and angles flames forward to ignite fuels^77^. Wind also moves heat ahead of the fire where it preheats and dries fuels thus facilitating ignition^77^. Slope can compound the effect of wind on the spread of fire by further angling flames toward fuels^77^.

Models suggest that fire frequency recorded 1701–1750 is partially influenced by the number of trees sampled at each FSR site. However, this result does not detract from the main findings for three reasons. (1) We found moderate collinearity with number of trees sampled versus temperature and versus elevation, meaning that number of trees may be an unintentional proxy for two predictors with meaningful relationships with fire frequency (i.e., higher fire frequency in warmer, low-elevation areas). (2) Predictors described above (i.e., distance to nearest trail, mean distance to nearest town) remained significant in models even when weighting observations by number of trees during model training (Supplementary Tables S1 and S2). (3) Models with number of trees as a predictor still showed significant relationships between fire frequency and distance to nearest trail, mean distance to nearest town, and mean wind speed.

Our models are most applicable to describing areas 0–10 km from the nearest trail, and 32–96 km (mean) from nearest town (Table 2). Our spatial predictions (Figs. 4 and 5) should be interpreted in a general sense due to low sample size, and because models extrapolated fire frequency at many locations within central PA. Models were trained with data from FSRs in more rugged, windier, and cooler upland conditions farther from towns and closer to trails. Further FSR development in areas closer to past towns (see next section) and farther from travel routes, in addition to testing the effect of proximity to other features of Indigenous settlement such as camps and cemeteries^79^, would refine correlative models of where fire occurred. Fire frequency is potentially higher than those modeled based on FSRs, because historical accounts suggest Indigenous peoples burned annually^70,74,80^, and because fires may not scar trees during low-intensity annual fires^23^. Finally, the 1701–1750 period is a post-Contact era of Indigenous land use, and therefore our models did not capture pre-Contact era fire practices.

### FSR locations versus Native American settlement

FSRs are far from areas of 18th- and 19th-century Native American settlement (Fig. 1), but based on the analysis of central PA, the current network of FSRs appears to record burning along trails. Higher trail density relative to town density, evidenced by various trail maps in the eastern US^81–85^, partially drives this result in PA and beyond: FSRs are more likely to be near trails than towns simply by chance. Burning captured in FSRs may have been associated with clearance of travel corridors, hunting, maintenance around non-residential sites, and management of fire-dependent communities rather than with purposes closer to towns such as agricultural clearing.

Locating trees that yield FSRs predating Euro-American settlement is challenging in eastern North America, and near Native American towns, due to various reasons: past tree harvesting, limited tree longevity before decay, and the need to develop FSRs from tree stumps or cross-sections versus core samples. Former Native American settlement in flatter terrain and near waterbodies appears to coincide with high-density modern development, further limiting available trees for sampling near past settlement. For instance, the southern Great Lakes region hosted numerous towns circa 1760–1810 (Fig. 1a–b), yet these areas appear to be heavily cleared today. The requirements of developing FSRs in eastern North America (i.e., old trees in environmentally-stressed locations less affected by modern land use) have likely led researchers to develop FSRs in marginal lands far from past settlement centers.

Characteristics of Native American land use further limit the possibility of discovering fire-scarred trees or tree remnants close to towns. Native Americans cleared large areas near towns to obtain building materials and create agricultural fields, and used fire near towns to promote berry and nut production^13,69,70^. Fire-scarred veteran trees near settlements likely did not survive into today. Creation of grasslands and savannas with few trees via burning would further limit the number of fire-scarred trees near past settlements. However, one dendrochronology study of growth releases in remnant >400-yr old white oak trees located 2 km from an Iroquois town site in northwestern PA estimated that releases occurred every 11 years during Native American occupation purportedly due to burning, because fire scars were observed^86^.

Sources used for mapping Native American settlement^35,36,45,60,61^ performed a detailed synthesis of various historical and other materials (Fig. 1), but the maps are still incomplete representations of where settlement occurred, an issue which affects our comparisons with FSR locations. More extensive data collection, including for non-residential sites, would refine understanding as to what types of burning (e.g. for hunting, nut production, agricultural clearing) are captured by FSRs.

### Improving geographic representation of Native American fire regimes via FSR site selection

This study demonstrates the importance of incorporating Native American geography for local- to landscape-scale understanding of fire frequency, and when searching for FSR sites so that such understanding can be advanced. Regional-extent sampling should occur in areas closer to Native American settlement regions in eastern North America (Fig. 1), and landscape-extent sampling closer to town or other sites, where older forests exist. It reinforces calls for more systematic sampling of eastern forests to determine the location and frequency of cultural burning^30^. Studies from the western US targeting locations of varying past Indigenous population density^87^ may serve as templates for efforts in eastern North America.

Expanding the FSR network^22^ and tree-ring sites more broadly^88^ is a time-sensitive issue, because trees and data they possess are being lost to mortality. Locating survivor trees is challenging due to aforementioned reasons related to Native American and Euro-American land-use history. Nevertheless, researchers have used modeling to predict locations of old-growth forests^89,90^, estimate forest age^37,91^, and locate “ancient” trees^92^. Further overlaying such predictions with accessible lands may refine where FSRs can be developed near past settlement. Moreover, Pederson^93^ provides practical advice on identifying deciduous trees ≥250 years old. Worth noting is that a recent paper discovered red pine (*Pinus resinosa*) tree stumps with annual rings dating to 1370 just 30 km from an 18th-century Native American town site and a few km from a travel corridor^62^.

Datasets on archaeological sites, or publications on Native American towns or travel routes, exist for much of eastern North America, which could be incorporated into FSR site planning and FSR-based modeling studies. Finer-resolution records of Native American sites can be gleaned from sources such as state historic preservation offices, museum records, and published literature^79^. For coarser-resolution maps or data, examples include Contact-era sites^94^, population density circa 1500 CE^95^, prehistoric settlement phases circa 900–1540 CE^72^, gridded Holocene population estimates^96^, and radiocarbon-dated archaeological sites^97^. Aside from those used here^35,36^, other map atlases on Native American history exist. Work in the eastern US has mapped trails for locations such as Ohio^82^, New York^81^, New England^85^, and the southeastern US^84^. As an alternative means of representing human mobility, modeling trail locations may present a means of comparing hypothetical travel routes^98^ with FSR-based fire frequency. Furthermore, we echo previous calls stating that physical scientists should seek to engage with archaeologists and Indigenous possessors of traditional knowledge in participatory research and collaboratives^99–101^. Such collaboration may lead to new hypotheses to test regarding the location of past cultural burning, in turn influencing the selection of new FSR sites.

## Conclusion

This study suggests that Indigenous burning occurred along trails and nearer to towns, and was further promoted by warmer temperatures and pyrophilic vegetation. Native American burning was a likely means of widespread alteration of forest composition that promoted fire-dependent species in PA. This study also revealed that FSRs are distant from past Native American towns, but close to past travel corridors. To further disentangle environmental from anthropogenic influences upon past fire regimes and forest conditions, FSR-based research in eastern North America should incorporate Indigenous geography to choose new sites for FSR development, ascertain fire regimes closer to former settlements, quantify the geographic extent of past cultural burning, and develop models of where burning occurred.

### Supplementary Information

Supplementary Information.

## Data Availability

Data used or created in this study, including GIS-format data layers on approximate Native American town and trail locations, are available from the corresponding author upon request. Various datasets used in this study are publicly available online.
