# Supplementary material for: Native American geography shaped historical fire frequency in forests of eighteenth-century Pennsylvania, USA
Source: Sci Rep. 2023 Oct 30;13:18598. doi: 10.1038/s41598-023-44692-5 (PMC10616284; doi:10.1038/s41598-023-44692-5)
Supplement: Supplementary file 1 — Supplementary Information. [file 41598_2023_44692_MOESM1_ESM.docx]

**Supplementary Information for article**

Native American geography shaped historical fire frequency in forests of 18^th^-century Pennsylvania, USA

Stephen J. Tulowiecki

Brice B. Hanberry

Marc D. Abrams

**Table S1.** All selected univariate and bivariate linear regression models, sorted first by weighting scheme and then by Akaike Information Criterion (AIC)

| **Weighting scheme** | **AIC** | ***R^2^*** | **Intercept or predictor** | **Coeff.** | **S.E.** | ***t*-stat** | ***p*-value** |
| --- | --- | --- | --- | --- | --- | --- | --- |
|  |  |  | intercept | -10.70 | 3.51 | -3.05 | 0.007 |
| *(unweighted)* | 99.0 | 0.653 | Distance to nearest trail | -0.45 | 0.20 | -2.28 | 0.035 |
|  |  |  | Mean annual temperature | 2.06 | 0.42 | 4.93 | <0.001 |
|  |  |  | intercept | -5.68 | 5.13 | -1.11 | 0.283 |
| *(unweighted)* | 100.1 | 0.634 | Mean distance to nearest town | -0.08 | 0.04 | -2.01 | 0.059 |
|  |  |  | Mean annual temperature | 1.86 | 0.46 | 4.06 | 0.001 |
|  |  |  | intercept | -13.44 | 3.64 | -3.69 | 0.002 |
| *(unweighted)* | 102.4 | 0.552 | Mean annual temperature | 2.21 | 0.46 | 4.84 | <0.001 |
|  |  |  | intercept | 16.74 | 4.59 | 3.65 | 0.002 |
| *(unweighted)* | 111.1 | 0.382 | Distance to nearest trail | -0.88 | 0.28 | -3.09 | 0.006 |
|  |  |  | Mean TRI | -0.66 | 0.28 | -2.40 | 0.028 |
|  |  |  | intercept | 10.00 | 2.06 | 4.84 | <0.001 |
| *(unweighted)* | 111.7 | 0.366 | Distance to nearest trail | -0.61 | 0.26 | -2.31 | 0.033 |
|  |  |  | Mean VRM | -917.17 | 404.22 | -2.27 | 0.036 |
|  |  |  | intercept | 12.82 | 3.17 | 4.05 | 0.001 |
| *(unweighted)* | 111.7 | 0.300 | Mean distance to nearest town | -0.14 | 0.05 | -2.85 | 0.010 |
|  |  |  | intercept | 2.96 | 2.02 | 1.46 | 0.161 |
| *(unweighted)* | 113.1 | 0.320 | Distance to nearest trail | -0.53 | 0.27 | -1.94 | 0.068 |
|  |  |  | Mean wind speed | 0.76 | 0.40 | 1.89 | 0.074 |
|  |  |  | intercept | 9.95 | 5.49 | 1.81 | 0.087 |
| *(unweighted)* | 113.8 | 0.299 | Elevation | -0.02 | 0.01 | -1.76 | 0.095 |
|  |  |  | Mean wind speed | 0.95 | 0.40 | 2.35 | 0.030 |
|  |  |  | intercept | -9.58 | 3.78 | -2.54 | 0.021 |
| by number of trees | 104.3 | 0.666 | Distance to nearest trail | -0.48 | 0.20 | -2.37 | 0.029 |
|  |  |  | Mean annual temperature | 1.96 | 0.42 | 4.62 | <0.001 |
|  |  |  | intercept | -5.84 | 5.27 | -1.11 | 0.282 |
| by number of trees | 105.9 | 0.640 | Mean distance to nearest town | -0.08 | 0.04 | -1.97 | 0.065 |
|  |  |  | Mean annual temperature | 1.85 | 0.47 | 3.98 | 0.001 |
|  |  |  | intercept | -13.58 | 3.76 | -3.61 | 0.002 |
| by number of trees | 108.0 | 0.562 | Mean annual temperature | 2.24 | 0.45 | 4.94 | <0.001 |
|  |  |  | intercept | 3.47 | 1.97 | 1.76 | 0.095 |
| by number of trees | 114.8 | 0.450 | Distance to nearest trail | -0.67 | 0.25 | -2.64 | 0.017 |
|  |  |  | Mean wind speed | 0.97 | 0.40 | 2.43 | 0.026 |
|  |  |  | intercept | 11.13 | 1.92 | 5.81 | <0.001 |
| by number of trees | 114.9 | 0.447 | Distance to nearest trail | -0.72 | 0.25 | -2.86 | 0.011 |
|  |  |  | Mean VRM | -930.73 | 388.24 | -2.40 | 0.028 |
|  |  |  | intercept | 16.73 | 4.32 | 3.87 | 0.001 |
| by number of trees | 115.6 | 0.429 | Distance to nearest trail | -0.94 | 0.27 | -3.48 | 0.003 |
|  |  |  | Mean TRI | -0.61 | 0.27 | -2.24 | 0.038 |
|  |  |  | intercept | 13.25 | 2.91 | 4.56 | <0.001 |
| by number of trees | 117.1 | 0.323 | Mean distance to nearest town | -0.14 | 0.05 | -3.01 | 0.007 |
|  |  |  | intercept | 9.55 | 5.27 | 1.81 | 0.087 |
| by number of trees | 118.2 | 0.354 | Elevation | -0.02 | 0.01 | -1.80 | 0.089 |
|  |  |  | Mean wind speed | 1.14 | 0.43 | 2.66 | 0.016 |
|  |  |  | intercept | 7.41 | 1.25 | 5.91 | <0.001 |
| by number of trees | 118.7 | 0.270 | Distance to nearest trail | -0.75 | 0.28 | -2.65 | 0.016 |
|  |  |  | intercept | 0.61 | 1.89 | 0.32 | 0.750 |
| by number of trees | 119.6 | 0.237 | Mean wind speed | 1.11 | 0.45 | 2.43 | 0.025 |
|  |  |  | intercept | 8.80 | 2.03 | 4.33 | <0.001 |
| by number of trees | 120.8 | 0.196 | Mean VRM | -979.35 | 455.15 | -2.15 | 0.044 |
|  |  |  | intercept | -18.25 | 2.54 | -7.19 | <0.001 |
| by area sampled | 96.0 | 0.859 | Mean annual temperature | 2.35 | 0.35 | 6.81 | <0.001 |
|  |  |  | Mean wind speed | 0.94 | 0.24 | 3.98 | 0.001 |
|  |  |  | intercept | -18.57 | 2.99 | -6.22 | <0.001 |
| by area sampled | 102.7 | 0.806 | Mean annual temperature | 2.63 | 0.39 | 6.84 | <0.001 |
|  |  |  | VRM | 220.28 | 86.10 | 2.56 | 0.020 |
|  |  |  | intercept | -15.12 | 3.75 | -4.04 | 0.001 |
| by area sampled | 104.2 | 0.792 | Distance to nearest trail | -0.32 | 0.15 | -2.21 | 0.040 |
|  |  |  | Mean annual temperature | 2.56 | 0.42 | 6.13 | <0.001 |
|  |  |  | intercept | -11.68 | 5.18 | -2.25 | 0.037 |
| by area sampled | 105.1 | 0.783 | Mean distance to nearest town | -0.06 | 0.03 | -2.00 | 0.061 |
|  |  |  | Mean annual temperature | 2.41 | 0.48 | 5.08 | <0.001 |
|  |  |  | intercept | -19.96 | 3.34 | -5.99 | <0.001 |
| by area sampled | 107.2 | 0.735 | Mean annual temperature | 2.98 | 0.41 | 7.27 | <0.001 |
|  |  |  | intercept | 5.71 | 4.02 | 1.42 | 0.173 |
| by area sampled | 118.5 | 0.590 | Mean distance to nearest town | -0.08 | 0.04 | -2.03 | 0.057 |
|  |  |  | Mean wind speed | 1.07 | 0.47 | 2.27 | 0.036 |
|  |  |  | intercept | 3.25 | 3.29 | 0.99 | 0.336 |
| by area sampled | 119.5 | 0.570 | Mean VRM | -1008.32 | 573.02 | -1.76 | 0.095 |
|  |  |  | Mean wind speed | 1.37 | 0.41 | 3.33 | 0.004 |
|  |  |  | intercept | 8.33 | 2.43 | 3.43 | 0.003 |
| by area sampled | 120.2 | 0.554 | VRM | 389.61 | 122.75 | 3.17 | 0.005 |
|  |  |  | Mean VRM | -1684.58 | 526.82 | -3.20 | 0.005 |
|  |  |  | intercept | -1.93 | 1.54 | -1.25 | 0.226 |
| by area sampled | 120.8 | 0.496 | Mean wind speed | 1.68 | 0.39 | 4.32 | <0.001 |
|  |  |  | intercept | 13.39 | 2.40 | 5.59 | <0.001 |
| by area sampled | 121.7 | 0.473 | Mean distance to nearest town | -0.14 | 0.03 | -4.13 | 0.001 |
|  |  |  | intercept | 11.77 | 2.39 | 4.92 | <0.001 |
| by area sampled | 123.2 | 0.487 | Distance to nearest trail | -0.55 | 0.22 | -2.52 | 0.021 |
|  |  |  | Mean VRM | -1288.53 | 604.07 | -2.13 | 0.047 |
|  |  |  | intercept | 7.37 | 1.32 | 5.59 | <0.001 |
| by area sampled | 125.9 | 0.357 | Distance to nearest trail | -0.72 | 0.22 | -3.25 | 0.004 |
|  |  |  | intercept | 11.41 | 2.71 | 4.22 | <0.001 |
| by area sampled | 127.5 | 0.305 | Mean VRM | -1840.84 | 637.57 | -2.89 | 0.009 |
|  |  |  | intercept | 1.31 | 1.26 | 1.04 | 0.311 |
| by area sampled | 127.6 | 0.301 | VRM | 426.29 | 148.96 | 2.86 | 0.010 |
|  |  |  | intercept | -8.80 | 4.02 | -2.19 | 0.042 |
| spatial weights | 114.1 | 0.705 | Distance to nearest trail | -0.57 | 0.20 | -2.90 | 0.010 |
|  |  |  | Mean annual temperature | 1.91 | 0.43 | 4.43 | <0.001 |
|  |  |  | intercept | -14.43 | 3.70 | -3.90 | 0.001 |
| spatial weights | 117.4 | 0.656 | Mean annual temperature | 1.93 | 0.48 | 4.04 | 0.001 |
|  |  |  | Mean wind speed | 0.82 | 0.38 | 2.15 | 0.045 |
|  |  |  | intercept | -5.00 | 6.06 | -0.83 | 0.420 |
| spatial weights | 117.7 | 0.651 | Mean distance to nearest town | -0.08 | 0.04 | -2.07 | 0.053 |
|  |  |  | Mean annual temperature | 1.79 | 0.51 | 3.48 | 0.003 |
|  |  |  | intercept | -14.91 | 4.03 | -3.70 | 0.002 |
| spatial weights | 120.2 | 0.567 | Mean annual temperature | 2.36 | 0.47 | 4.99 | <0.001 |
|  |  |  | intercept | 3.40 | 1.99 | 1.71 | 0.105 |
| spatial weights | 120.7 | 0.596 | Distance to nearest trail | -0.73 | 0.22 | -3.35 | 0.004 |
|  |  |  | Mean wind speed | 1.18 | 0.38 | 3.08 | 0.006 |
|  |  |  | intercept | 11.87 | 1.65 | 7.20 | <0.001 |
| spatial weights | 122.3 | 0.564 | Distance to nearest trail | -0.85 | 0.22 | -3.85 | 0.001 |
|  |  |  | Mean VRM | -844.13 | 308.48 | -2.74 | 0.014 |
|  |  |  | intercept | 11.34 | 1.86 | 6.11 | <0.001 |
| spatial weights | 125.5 | 0.493 | Distance to nearest trail | -0.94 | 0.24 | -3.92 | 0.001 |
|  |  |  | TRI | -0.14 | 0.07 | -1.98 | 0.064 |
|  |  |  | intercept | 15.76 | 4.25 | 3.71 | 0.002 |
| spatial weights | 126.2 | 0.475 | Distance to nearest trail | -1.02 | 0.26 | -3.99 | 0.001 |
|  |  |  | Mean TRI | -0.50 | 0.28 | -1.77 | 0.093 |
|  |  |  | intercept | 14.68 | 2.74 | 5.35 | <0.001 |
| spatial weights | 126.5 | 0.416 | Mean distance to nearest town | -0.15 | 0.04 | -3.68 | 0.002 |
|  |  |  | intercept | 8.55 | 1.29 | 6.61 | <0.001 |
| spatial weights | 127.6 | 0.383 | Distance to nearest trail | -0.88 | 0.26 | -3.43 | 0.003 |
|  |  |  | intercept | -0.67 | 1.96 | -0.34 | 0.737 |
| spatial weights | 128.9 | 0.344 | Mean wind speed | 1.46 | 0.46 | 3.16 | 0.005 |
|  |  |  | intercept | 6.17 | 1.95 | 3.17 | 0.005 |
| spatial weights | 129.0 | 0.401 | VRM | 386.66 | 159.42 | 2.43 | 0.026 |
|  |  |  | Mean VRM | -938.27 | 361.79 | -2.59 | 0.018 |
|  |  |  | intercept | -0.39 | 4.68 | -0.08 | 0.934 |
| spatial weights | 130.5 | 0.358 | Mean TRI | 0.96 | 0.46 | 2.07 | 0.053 |
|  |  |  | Mean VRM | -1832.26 | 586.96 | -3.12 | 0.006 |
|  |  |  | intercept | 4.97 | 1.77 | 2.81 | 0.012 |
| spatial weights | 130.5 | 0.357 | TRI | -0.19 | 0.09 | -2.24 | 0.038 |
|  |  |  | VRM | 506.98 | 176.43 | 2.87 | 0.010 |
|  |  |  | intercept | 8.62 | 1.86 | 4.62 | <0.001 |
| spatial weights | 133.0 | 0.205 | Mean VRM | -896.65 | 405.17 | -2.21 | 0.039 |

**Table S2.** All univariate and bivariate Poisson regression models, sorted first by weighting scheme and then by Akaike Information Criterion (AIC)

| **Weighting scheme** | **AIC** | **R^2^** | **Intercept or predictor** | **Coeff.** | **S.E.** | ***t*-stat** | **p-value** |
| --- | --- | --- | --- | --- | --- | --- | --- |
|  |  |  | intercept | -1.79 | 0.80 | -2.23 | 0.026 |
| *(unweighted)* | 88.1 | 0.631 | Distance to nearest trail | -0.12 | 0.05 | -2.31 | 0.021 |
|  |  |  | Mean annual temperature | 0.43 | 0.09 | 4.96 | <0.001 |
|  |  |  | intercept | -1.02 | 1.09 | -0.94 | 0.348 |
| *(unweighted)* | 90.3 | 0.596 | Mean distance to nearest town | -0.02 | 0.01 | -1.99 | 0.047 |
|  |  |  | Mean annual temperature | 0.40 | 0.09 | 4.28 | <0.001 |
|  |  |  | intercept | -2.59 | 0.73 | -3.53 | <0.001 |
| *(unweighted)* | 92.4 | 0.530 | Mean annual temperature | 0.48 | 0.08 | 5.76 | <0.001 |
|  |  |  | intercept | 3.03 | 0.34 | 8.96 | <0.001 |
| *(unweighted)* | 99.6 | 0.447 | Distance to nearest trail | -0.18 | 0.05 | -3.36 | <0.001 |
|  |  |  | Mean VRM | -284.96 | 82.58 | -3.45 | <0.001 |
|  |  |  | intercept | 4.17 | 0.68 | 6.14 | <0.001 |
| *(unweighted)* | 102.1 | 0.408 | Distance to nearest trail | -0.23 | 0.05 | -4.27 | <0.001 |
|  |  |  | Mean TRI | -0.15 | 0.05 | -3.24 | 0.001 |
|  |  |  | intercept | 3.67 | 0.45 | 8.07 | <0.001 |
| *(unweighted)* | 103.3 | 0.389 | Mean distance to nearest town | -0.03 | 0.01 | -3.19 | 0.001 |
|  |  |  | Mean VRM | -181.80 | 88.96 | -2.04 | 0.041 |
|  |  |  | intercept | -1.62 | 1.23 | -1.33 | 0.185 |
| *(unweighted)* | 103.8 | 0.382 | Distance to nearest trail | -0.29 | 0.07 | -4.13 | <0.001 |
|  |  |  | Mean annual precipitation | 0.00 | 0.00 | 2.99 | 0.003 |
|  |  |  | intercept | 3.56 | 0.49 | 7.34 | <0.001 |
| *(unweighted)* | 104.5 | 0.370 | Mean distance to nearest town | -0.05 | 0.01 | -4.07 | <0.001 |
|  |  |  | Distance to nearest 6th-order stream | 0.04 | 0.03 | 1.67 | 0.094 |
|  |  |  | intercept | 1.14 | 0.35 | 3.23 | 0.001 |
| *(unweighted)* | 104.6 | 0.368 | Distance to nearest trail | -0.17 | 0.06 | -3.05 | 0.002 |
|  |  |  | Mean wind speed | 0.18 | 0.06 | 2.84 | 0.005 |
|  |  |  | intercept | 3.24 | 0.46 | 7.09 | <0.001 |
| *(unweighted)* | 104.7 | 0.367 | Mean distance to nearest town | -0.03 | 0.01 | -2.82 | 0.005 |
|  |  |  | Distance to nearest trail | -0.11 | 0.07 | -1.67 | 0.095 |
|  |  |  | intercept | 2.58 | 0.67 | 3.86 | <0.001 |
| *(unweighted)* | 104.7 | 0.366 | Mean distance to nearest town | -0.03 | 0.01 | -3.36 | <0.001 |
|  |  |  | Mean wind speed | 0.11 | 0.07 | 1.70 | 0.090 |
|  |  |  | intercept | 2.73 | 0.32 | 8.48 | <0.001 |
| *(unweighted)* | 104.8 | 0.364 | Distance to nearest trail | -0.21 | 0.06 | -3.80 | <0.001 |
|  |  |  | TRI | -0.04 | 0.01 | -2.74 | 0.006 |
|  |  |  | intercept | 3.44 | 0.44 | 7.84 | <0.001 |
| *(unweighted)* | 105.6 | 0.320 | Mean distance to nearest town | -0.04 | 0.01 | -4.55 | <0.001 |
|  |  |  | intercept | 4.13 | 0.76 | 5.47 | <0.001 |
| *(unweighted)* | 108.6 | 0.305 | Mean VRM | -285.40 | 82.78 | -3.45 | <0.001 |
|  |  |  | Elevation | 0.00 | 0.00 | -2.28 | 0.023 |
|  |  |  | intercept | 1.61 | 0.51 | 3.13 | 0.002 |
| *(unweighted)* | 108.9 | 0.299 | Mean VRM | -213.06 | 84.48 | -2.52 | 0.012 |
|  |  |  | Mean wind speed | 0.15 | 0.07 | 2.18 | 0.030 |
|  |  |  | intercept | 2.30 | 0.77 | 2.99 | 0.003 |
| *(unweighted)* | 109.7 | 0.287 | Elevation | 0.00 | 0.00 | -2.53 | 0.012 |
|  |  |  | Mean wind speed | 0.21 | 0.06 | 3.52 | <0.001 |
|  |  |  | intercept | 1.98 | 0.18 | 10.92 | <0.001 |
| *(unweighted)* | 110.7 | 0.239 | Distance to nearest trail | -0.20 | 0.06 | -3.46 | <0.001 |
|  |  |  | intercept | 2.51 | 0.31 | 8.00 | <0.001 |
| *(unweighted)* | 111.7 | 0.224 | Mean VRM | -286.17 | 81.09 | -3.53 | <0.001 |
|  |  |  | intercept | 0.51 | 0.30 | 1.69 | 0.090 |
| *(unweighted)* | 113.9 | 0.189 | Mean wind speed | 0.21 | 0.06 | 3.42 | <0.001 |
|  |  |  | intercept | 3.87 | 0.79 | 4.93 | <0.001 |
| *(unweighted)* | 116.3 | 0.181 | TRI | -0.03 | 0.01 | -2.40 | 0.016 |
|  |  |  | Elevation | 0.00 | 0.00 | -2.59 | 0.010 |
|  |  |  | intercept | 3.23 | 0.77 | 4.18 | <0.001 |
| *(unweighted)* | 120.3 | 0.087 | Elevation | 0.00 | 0.00 | -2.37 | 0.018 |
|  |  |  | intercept | 1.88 | 0.24 | 7.87 | <0.001 |
| *(unweighted)* | 120.8 | 0.079 | TRI | -0.03 | 0.01 | -2.20 | 0.028 |
|  |  |  | intercept | 2.46 | 0.62 | 3.94 | <0.001 |
| *(unweighted)* | 121.4 | 0.101 | VRM | 31.23 | 18.24 | 1.71 | 0.087 |
|  |  |  | Mean TRI | -0.09 | 0.04 | -2.11 | 0.035 |
|  |  |  | intercept | -1.42 | 1.40 | -1.01 | 0.311 |
| *(unweighted)* | 122.4 | 0.085 | Mean annual precipitation | 0.00 | 0.00 | 1.89 | 0.060 |
|  |  |  | VRM | 44.18 | 20.81 | 2.12 | 0.034 |
|  |  |  | intercept | -1.50 | 0.16 | -9.49 | <0.001 |
| by number of trees | 2382.5 | 0.672 | Distance to nearest trail | -0.12 | 0.01 | -12.67 | <0.001 |
|  |  |  | Mean annual temperature | 0.40 | 0.02 | 24.05 | <0.001 |
|  |  |  | intercept | -1.18 | 0.19 | -6.09 | <0.001 |
| by number of trees | 2463.0 | 0.628 | Mean distance to nearest town | -0.01 | 0.00 | -10.35 | <0.001 |
|  |  |  | Mean annual temperature | 0.40 | 0.02 | 23.43 | <0.001 |
|  |  |  | intercept | -4.44 | 0.28 | -15.82 | <0.001 |
| by number of trees | 2505.0 | 0.606 | Mean annual temperature | 0.56 | 0.02 | 29.47 | <0.001 |
|  |  |  | Mean TRI | 0.09 | 0.01 | 8.19 | <0.001 |
|  |  |  | intercept | -2.38 | 0.14 | -17.07 | <0.001 |
| by number of trees | 2522.1 | 0.596 | Mean annual temperature | 0.42 | 0.02 | 24.34 | <0.001 |
|  |  |  | Mean wind speed | 0.08 | 0.01 | 7.16 | <0.001 |
|  |  |  | intercept | -2.54 | 0.14 | -18.24 | <0.001 |
| by number of trees | 2538.5 | 0.587 | Mean annual temperature | 0.46 | 0.02 | 29.13 | <0.001 |
|  |  |  | VRM | 20.76 | 3.42 | 6.07 | <0.001 |
|  |  |  | intercept | -3.23 | 0.24 | -13.37 | <0.001 |
| by number of trees | 2561.5 | 0.575 | Mean annual temperature | 0.53 | 0.02 | 24.43 | <0.001 |
|  |  |  | Mean VRM | 62.07 | 17.16 | 3.62 | <0.001 |
|  |  |  | intercept | -2.28 | 0.16 | -14.44 | <0.001 |
| by number of trees | 2562.9 | 0.574 | Mean annual temperature | 0.46 | 0.02 | 29.28 | <0.001 |
|  |  |  | TRI | -0.01 | 0.00 | -3.37 | <0.001 |
|  |  |  | intercept | -1.77 | 0.27 | -6.47 | <0.001 |
| by number of trees | 2563.8 | 0.574 | Mean annual temperature | 0.48 | 0.02 | 30.87 | <0.001 |
|  |  |  | Mean annual precipitation | 0.00 | 0.00 | -3.21 | 0.001 |
|  |  |  | intercept | -2.53 | 0.14 | -17.98 | <0.001 |
| by number of trees | 2572.3 | 0.568 | Mean annual temperature | 0.48 | 0.02 | 30.70 | <0.001 |
|  |  |  | intercept | 2.99 | 0.05 | 56.01 | <0.001 |
| by number of trees | 2671.1 | 0.516 | Distance to nearest trail | -0.19 | 0.01 | -20.16 | <0.001 |
|  |  |  | Mean VRM | -237.05 | 13.82 | -17.16 | <0.001 |
|  |  |  | intercept | 1.34 | 0.06 | 22.27 | <0.001 |
| by number of trees | 2714.7 | 0.492 | Distance to nearest trail | -0.18 | 0.01 | -19.44 | <0.001 |
|  |  |  | Mean wind speed | 0.18 | 0.01 | 16.95 | <0.001 |
|  |  |  | intercept | 3.51 | 0.08 | 45.57 | <0.001 |
| by number of trees | 2762.8 | 0.466 | Mean distance to nearest town | -0.05 | 0.00 | -23.76 | <0.001 |
|  |  |  | Distance to nearest 6th-order stream | 0.06 | 0.00 | 14.77 | <0.001 |
|  |  |  | intercept | 3.76 | 0.11 | 34.05 | <0.001 |
| by number of trees | 2785.1 | 0.454 | Distance to nearest trail | -0.22 | 0.01 | -24.38 | <0.001 |
|  |  |  | Mean TRI | -0.11 | 0.01 | -14.43 | <0.001 |
|  |  |  | intercept | 2.77 | 0.05 | 51.94 | <0.001 |
| by number of trees | 2829.0 | 0.430 | Distance to nearest trail | -0.22 | 0.01 | -23.20 | <0.001 |
|  |  |  | TRI | -0.03 | 0.00 | -12.94 | <0.001 |
|  |  |  | intercept | 2.99 | 0.07 | 45.03 | <0.001 |
| by number of trees | 2830.9 | 0.429 | Mean distance to nearest town | -0.02 | 0.00 | -12.84 | <0.001 |
|  |  |  | Distance to nearest trail | -0.15 | 0.01 | -13.36 | <0.001 |
|  |  |  | intercept | -0.23 | 0.23 | -1.00 | 0.316 |
| by number of trees | 2890.5 | 0.396 | Distance to nearest trail | -0.26 | 0.01 | -23.12 | <0.001 |
|  |  |  | Mean annual precipitation | 0.00 | 0.00 | 10.49 | <0.001 |
|  |  |  | intercept | 3.43 | 0.07 | 49.67 | <0.001 |
| by number of trees | 2890.6 | 0.396 | Mean distance to nearest town | -0.02 | 0.00 | -17.04 | <0.001 |
|  |  |  | Mean VRM | -163.50 | 14.74 | -11.09 | <0.001 |
|  |  |  | intercept | 2.32 | 0.11 | 20.32 | <0.001 |
| by number of trees | 2928.4 | 0.376 | Mean distance to nearest town | -0.02 | 0.00 | -15.97 | <0.001 |
|  |  |  | Mean wind speed | 0.12 | 0.01 | 9.57 | <0.001 |
|  |  |  | intercept | 2.10 | 0.03 | 62.84 | <0.001 |
| by number of trees | 2971.7 | 0.352 | Distance to nearest trail | -0.24 | 0.01 | -21.14 | <0.001 |
|  |  |  | Distance to nearest 5th-order stream | 0.04 | 0.01 | 5.67 | <0.001 |
|  |  |  | intercept | 2.00 | 0.05 | 39.58 | <0.001 |
| by number of trees | 2983.1 | 0.346 | Distance to nearest trail | -0.20 | 0.01 | -20.13 | <0.001 |
|  |  |  | VRM | 17.14 | 3.83 | 4.48 | <0.001 |
|  |  |  | intercept | 2.54 | 0.13 | 20.23 | <0.001 |
| by number of trees | 2993.9 | 0.340 | Distance to nearest trail | -0.20 | 0.01 | -18.30 | <0.001 |
|  |  |  | Elevation | 0.00 | 0.00 | -2.91 | 0.004 |
|  |  |  | intercept | 2.25 | 0.04 | 51.75 | <0.001 |
| by number of trees | 2997.9 | 0.338 | Distance to nearest trail | -0.22 | 0.01 | -22.04 | <0.001 |
|  |  |  | Distance to nearest 6th-order stream | -0.01 | 0.00 | -2.11 | 0.035 |
|  |  |  | intercept | 2.18 | 0.03 | 73.06 | <0.001 |
| by number of trees | 3000.4 | 0.336 | Distance to nearest trail | -0.22 | 0.01 | -21.95 | <0.001 |
|  |  |  | intercept | 3.56 | 0.11 | 31.75 | <0.001 |
| by number of trees | 3006.4 | 0.334 | Mean distance to nearest town | -0.03 | 0.00 | -19.17 | <0.001 |
|  |  |  | Elevation | 0.00 | 0.00 | -3.83 | <0.001 |
|  |  |  | intercept | 3.27 | 0.07 | 48.19 | <0.001 |
| by number of trees | 3007.3 | 0.333 | Mean distance to nearest town | -0.03 | 0.00 | -21.91 | <0.001 |
|  |  |  | TRI | -0.01 | 0.00 | -3.73 | <0.001 |
|  |  |  | intercept | 1.57 | 0.09 | 17.91 | <0.001 |
| by number of trees | 3007.5 | 0.333 | Mean VRM | -178.41 | 14.08 | -12.68 | <0.001 |
|  |  |  | Mean wind speed | 0.16 | 0.01 | 13.54 | <0.001 |
|  |  |  | intercept | 3.47 | 0.11 | 33.00 | <0.001 |
| by number of trees | 3010.7 | 0.331 | Mean distance to nearest town | -0.03 | 0.00 | -21.77 | <0.001 |
|  |  |  | Mean TRI | -0.03 | 0.01 | -3.23 | 0.001 |
|  |  |  | intercept | 2.73 | 0.22 | 12.33 | <0.001 |
| by number of trees | 3016.2 | 0.328 | Mean distance to nearest town | -0.03 | 0.00 | -24.59 | <0.001 |
|  |  |  | Mean annual precipitation | 0.00 | 0.00 | 2.24 | 0.025 |
|  |  |  | intercept | 3.21 | 0.07 | 48.97 | <0.001 |
| by number of trees | 3019.2 | 0.326 | Mean distance to nearest town | -0.03 | 0.00 | -24.47 | <0.001 |
|  |  |  | intercept | 4.04 | 0.12 | 32.69 | <0.001 |
| by number of trees | 3023.0 | 0.325 | Mean VRM | -263.60 | 14.07 | -18.73 | <0.001 |
|  |  |  | Elevation | 0.00 | 0.00 | -12.81 | <0.001 |
|  |  |  | intercept | 2.26 | 0.06 | 39.65 | <0.001 |
| by number of trees | 3028.5 | 0.322 | VRM | 42.35 | 3.21 | 13.18 | <0.001 |
|  |  |  | Mean VRM | -282.46 | 14.26 | -19.80 | <0.001 |
|  |  |  | intercept | 2.11 | 0.13 | 16.28 | <0.001 |
| by number of trees | 3030.4 | 0.321 | Elevation | 0.00 | 0.00 | -12.36 | <0.001 |
|  |  |  | Mean wind speed | 0.21 | 0.01 | 19.98 | <0.001 |
|  |  |  | intercept | 0.26 | 0.07 | 3.63 | <0.001 |
| by number of trees | 3119.2 | 0.272 | Distance to nearest 6th-order stream | 0.02 | 0.00 | 8.21 | <0.001 |
|  |  |  | Mean wind speed | 0.25 | 0.01 | 22.00 | <0.001 |
|  |  |  | intercept | 1.82 | 0.11 | 15.83 | <0.001 |
| by number of trees | 3138.5 | 0.262 | Mean TRI | 0.08 | 0.01 | 7.07 | <0.001 |
|  |  |  | Mean VRM | -346.87 | 18.26 | -19.00 | <0.001 |
|  |  |  | intercept | 2.70 | 0.06 | 47.38 | <0.001 |
| by number of trees | 3157.0 | 0.252 | Distance to nearest 5th-order stream | -0.04 | 0.01 | -5.51 | <0.001 |
|  |  |  | Mean VRM | -267.80 | 13.52 | -19.80 | <0.001 |
|  |  |  | intercept | 0.82 | 0.08 | 10.45 | <0.001 |
| by number of trees | 3176.6 | 0.241 | TRI | -0.01 | 0.00 | -3.00 | 0.003 |
|  |  |  | Mean wind speed | 0.21 | 0.01 | 18.28 | <0.001 |
|  |  |  | intercept | 1.01 | 0.13 | 7.57 | <0.001 |
| by number of trees | 3176.6 | 0.241 | Mean TRI | -0.02 | 0.01 | -3.00 | 0.003 |
|  |  |  | Mean wind speed | 0.22 | 0.01 | 18.92 | <0.001 |
|  |  |  | intercept | 3.39 | 0.25 | 13.44 | <0.001 |
| by number of trees | 3177.3 | 0.241 | Mean annual precipitation | 0.00 | 0.00 | -3.43 | <0.001 |
|  |  |  | Mean VRM | -276.70 | 14.64 | -18.90 | <0.001 |
|  |  |  | intercept | 0.63 | 0.05 | 12.06 | <0.001 |
| by number of trees | 3180.8 | 0.239 | VRM | -8.78 | 3.98 | -2.21 | 0.027 |
|  |  |  | Mean wind speed | 0.24 | 0.01 | 17.49 | <0.001 |
|  |  |  | intercept | 0.19 | 0.23 | 0.84 | 0.403 |
| by number of trees | 3181.5 | 0.239 | Mean annual precipitation | 0.00 | 0.00 | 2.04 | 0.042 |
|  |  |  | Mean wind speed | 0.23 | 0.01 | 20.76 | <0.001 |
|  |  |  | intercept | 0.64 | 0.05 | 12.31 | <0.001 |
| by number of trees | 3183.7 | 0.236 | Mean wind speed | 0.23 | 0.01 | 20.80 | <0.001 |
|  |  |  | intercept | 2.55 | 0.05 | 50.40 | <0.001 |
| by number of trees | 3187.2 | 0.234 | Mean VRM | -261.30 | 13.54 | -19.30 | <0.001 |
|  |  |  | intercept | 3.74 | 0.13 | 29.23 | <0.001 |
| by number of trees | 3310.0 | 0.169 | TRI | -0.02 | 0.00 | -11.03 | <0.001 |
|  |  |  | Elevation | 0.00 | 0.00 | -14.35 | <0.001 |
|  |  |  | intercept | 1.69 | 0.05 | 34.64 | <0.001 |
| by number of trees | 3356.2 | 0.144 | TRI | -0.03 | 0.00 | -11.56 | <0.001 |
|  |  |  | VRM | 40.00 | 3.02 | 13.25 | <0.001 |
|  |  |  | intercept | 2.37 | 0.11 | 22.39 | <0.001 |
| by number of trees | 3367.0 | 0.138 | VRM | 42.87 | 3.17 | 13.51 | <0.001 |
|  |  |  | Mean TRI | -0.08 | 0.01 | -11.00 | <0.001 |
|  |  |  | intercept | 2.73 | 0.14 | 19.53 | <0.001 |
| by number of trees | 3370.3 | 0.136 | VRM | 28.97 | 3.52 | 8.24 | <0.001 |
|  |  |  | Elevation | 0.00 | 0.00 | -10.96 | <0.001 |
|  |  |  | intercept | 3.69 | 0.14 | 26.23 | <0.001 |
| by number of trees | 3396.8 | 0.122 | Mean TRI | -0.05 | 0.01 | -6.09 | <0.001 |
|  |  |  | Elevation | 0.00 | 0.00 | -11.56 | <0.001 |
|  |  |  | intercept | 3.24 | 0.13 | 25.73 | <0.001 |
| by number of trees | 3411.8 | 0.114 | Distance to nearest 6th-order stream | 0.01 | 0.00 | 4.84 | <0.001 |
|  |  |  | Elevation | 0.00 | 0.00 | -14.20 | <0.001 |
|  |  |  | intercept | 3.28 | 0.12 | 26.26 | <0.001 |
| by number of trees | 3432.5 | 0.101 | Elevation | 0.00 | 0.00 | -13.63 | <0.001 |
|  |  |  | intercept | -0.62 | 0.27 | -2.27 | 0.023 |
| by number of trees | 3445.3 | 0.096 | Mean annual precipitation | 0.00 | 0.00 | 6.92 | <0.001 |
|  |  |  | VRM | 51.92 | 3.77 | 13.76 | <0.001 |
|  |  |  | intercept | 2.22 | 0.06 | 39.93 | <0.001 |
| by number of trees | 3466.9 | 0.084 | Distance to nearest 5th-order stream | -0.05 | 0.01 | -6.81 | <0.001 |
|  |  |  | TRI | -0.03 | 0.00 | -11.76 | <0.001 |
|  |  |  | intercept | 1.84 | 0.04 | 40.99 | <0.001 |
| by number of trees | 3474.8 | 0.080 | Distance to nearest 6th-order stream | 0.02 | 0.00 | 6.47 | <0.001 |
|  |  |  | TRI | -0.03 | 0.00 | -11.61 | <0.001 |
|  |  |  | intercept | 1.14 | 0.05 | 23.35 | <0.001 |
| by number of trees | 3483.2 | 0.075 | Distance to nearest 6th-order stream | 0.01 | 0.00 | 3.21 | 0.001 |
|  |  |  | VRM | 39.16 | 3.29 | 11.91 | <0.001 |
|  |  |  | intercept | 1.24 | 0.04 | 35.34 | <0.001 |
| by number of trees | 3491.4 | 0.069 | VRM | 38.37 | 3.27 | 11.74 | <0.001 |
|  |  |  | intercept | 2.98 | 0.25 | 11.73 | <0.001 |
| by number of trees | 3499.2 | 0.066 | Mean annual precipitation | 0.00 | 0.00 | -4.10 | <0.001 |
|  |  |  | TRI | -0.03 | 0.00 | -10.72 | <0.001 |
|  |  |  | intercept | 2.29 | 0.11 | 20.95 | <0.001 |
| by number of trees | 3505.3 | 0.063 | TRI | -0.01 | 0.00 | -5.33 | <0.001 |
|  |  |  | Mean TRI | -0.03 | 0.01 | -3.30 | <0.001 |
|  |  |  | intercept | 2.72 | 0.11 | 24.90 | <0.001 |
| by number of trees | 3507.2 | 0.062 | Distance to nearest 5th-order stream | -0.03 | 0.01 | -5.07 | <0.001 |
|  |  |  | Mean TRI | -0.07 | 0.01 | -9.98 | <0.001 |
|  |  |  | intercept | 2.50 | 0.10 | 24.11 | <0.001 |
| by number of trees | 3507.7 | 0.062 | Distance to nearest 6th-order stream | 0.01 | 0.00 | 5.22 | <0.001 |
|  |  |  | Mean TRI | -0.08 | 0.01 | -10.11 | <0.001 |
|  |  |  | intercept | 1.95 | 0.04 | 47.87 | <0.001 |
| by number of trees | 3514.2 | 0.057 | TRI | -0.02 | 0.00 | -10.19 | <0.001 |
|  |  |  | intercept | 3.85 | 0.32 | 11.89 | <0.001 |
| by number of trees | 3514.4 | 0.058 | Mean annual precipitation | 0.00 | 0.00 | -4.40 | <0.001 |
|  |  |  | Mean TRI | -0.09 | 0.01 | -10.04 | <0.001 |
|  |  |  | intercept | 2.50 | 0.10 | 24.60 | <0.001 |
| by number of trees | 3532.3 | 0.047 | Mean TRI | -0.07 | 0.01 | -9.21 | <0.001 |
|  |  |  | intercept | 0.64 | 0.27 | 2.34 | 0.019 |
| by number of trees | 3595.8 | 0.014 | Distance to nearest 5th-order stream | -0.05 | 0.01 | -4.99 | <0.001 |
|  |  |  | Mean annual precipitation | 0.00 | 0.00 | 3.73 | <0.001 |
|  |  |  | intercept | 1.57 | 0.04 | 37.23 | <0.001 |
| by number of trees | 3602.0 | 0.011 | Distance to nearest 5th-order stream | -0.02 | 0.01 | -3.53 | <0.001 |
|  |  |  | Distance to nearest 6th-order stream | 0.01 | 0.00 | 2.79 | 0.005 |
|  |  |  | intercept | 1.65 | 0.03 | 53.60 | <0.001 |
| by number of trees | 3607.7 | 0.006 | Distance to nearest 5th-order stream | -0.02 | 0.01 | -3.38 | <0.001 |
|  |  |  | intercept | 1.49 | 0.04 | 41.61 | <0.001 |
| by number of trees | 3612.9 | 0.004 | Distance to nearest 6th-order stream | 0.01 | 0.00 | 2.58 | 0.010 |
|  |  |  | intercept | -2.95 | 0.16 | -18.37 | <0.001 |
| by area sampled | 3470.9 | 0.799 | Distance to nearest trail | -0.12 | 0.01 | -15.60 | <0.001 |
|  |  |  | Mean annual temperature | 0.55 | 0.02 | 34.01 | <0.001 |
|  |  |  | intercept | -4.04 | 0.13 | -31.54 | <0.001 |
| by area sampled | 3484.2 | 0.796 | Mean annual temperature | 0.55 | 0.02 | 32.93 | <0.001 |
|  |  |  | Mean wind speed | 0.17 | 0.01 | 15.84 | <0.001 |
|  |  |  | intercept | -6.91 | 0.21 | -33.19 | <0.001 |
| by area sampled | 3558.1 | 0.780 | Mean annual temperature | 0.76 | 0.01 | 53.50 | <0.001 |
|  |  |  | Mean TRI | 0.13 | 0.01 | 13.76 | <0.001 |
|  |  |  | intercept | -4.44 | 0.13 | -34.42 | <0.001 |
| by area sampled | 3583.2 | 0.774 | Mean annual temperature | 0.64 | 0.01 | 43.50 | <0.001 |
|  |  |  | VRM | 33.60 | 2.59 | 12.96 | <0.001 |
|  |  |  | intercept | -2.77 | 0.20 | -13.56 | <0.001 |
| by area sampled | 3583.9 | 0.774 | Mean distance to nearest town | -0.01 | 0.00 | -12.48 | <0.001 |
|  |  |  | Mean annual temperature | 0.57 | 0.02 | 31.98 | <0.001 |
|  |  |  | intercept | -2.26 | 0.25 | -9.06 | <0.001 |
| by area sampled | 3609.7 | 0.768 | Mean annual temperature | 0.74 | 0.01 | 53.85 | <0.001 |
|  |  |  | Mean annual precipitation | 0.00 | 0.00 | -11.63 | <0.001 |
|  |  |  | intercept | -4.68 | 0.13 | -36.31 | <0.001 |
| by area sampled | 3710.2 | 0.746 | Distance to nearest 5th-order stream | -0.03 | 0.01 | -5.85 | <0.001 |
|  |  |  | Mean annual temperature | 0.71 | 0.01 | 52.54 | <0.001 |
|  |  |  | intercept | -4.95 | 0.13 | -38.36 | <0.001 |
| by area sampled | 3716.1 | 0.745 | Distance to nearest 6th-order stream | 0.01 | 0.00 | 5.67 | <0.001 |
|  |  |  | Mean annual temperature | 0.72 | 0.01 | 52.42 | <0.001 |
|  |  |  | intercept | -5.77 | 0.22 | -26.48 | <0.001 |
| by area sampled | 3719.4 | 0.744 | Mean annual temperature | 0.79 | 0.02 | 41.72 | <0.001 |
|  |  |  | Mean VRM | 92.38 | 17.50 | 5.28 | <0.001 |
|  |  |  | intercept | -5.09 | 0.14 | -36.24 | <0.001 |
| by area sampled | 3726.4 | 0.742 | Mean annual temperature | 0.73 | 0.01 | 52.29 | <0.001 |
|  |  |  | TRI | 0.01 | 0.00 | 4.56 | <0.001 |
|  |  |  | intercept | -5.13 | 0.16 | -31.95 | <0.001 |
| by area sampled | 3738.4 | 0.740 | Mean annual temperature | 0.72 | 0.01 | 52.87 | <0.001 |
|  |  |  | Elevation | 0.00 | 0.00 | 2.93 | 0.003 |
|  |  |  | intercept | -4.84 | 0.13 | -38.04 | <0.001 |
| by area sampled | 3744.9 | 0.738 | Mean annual temperature | 0.72 | 0.01 | 52.57 | <0.001 |
|  |  |  | intercept | 0.90 | 0.06 | 13.91 | <0.001 |
| by area sampled | 4242.6 | 0.628 | Distance to nearest trail | -0.18 | 0.01 | -21.97 | <0.001 |
|  |  |  | Mean wind speed | 0.25 | 0.01 | 24.49 | <0.001 |
|  |  |  | intercept | 1.96 | 0.10 | 19.48 | <0.001 |
| by area sampled | 4253.1 | 0.625 | Mean distance to nearest town | -0.03 | 0.00 | -23.59 | <0.001 |
|  |  |  | Mean wind speed | 0.22 | 0.01 | 19.64 | <0.001 |
|  |  |  | intercept | 2.65 | 0.05 | 53.02 | <0.001 |
| by area sampled | 4345.8 | 0.605 | VRM | 84.72 | 2.36 | 35.88 | <0.001 |
|  |  |  | Mean VRM | -538.55 | 14.22 | -37.86 | <0.001 |
|  |  |  | intercept | 3.16 | 0.04 | 71.74 | <0.001 |
| by area sampled | 4398.0 | 0.593 | Distance to nearest trail | -0.23 | 0.01 | -29.09 | <0.001 |
|  |  |  | Mean VRM | -286.20 | 13.44 | -21.30 | <0.001 |
|  |  |  | intercept | 0.95 | 0.08 | 12.21 | <0.001 |
| by area sampled | 4411.4 | 0.590 | Mean VRM | -267.80 | 13.57 | -19.73 | <0.001 |
|  |  |  | Mean wind speed | 0.33 | 0.01 | 33.51 | <0.001 |
|  |  |  | intercept | 4.03 | 0.05 | 76.53 | <0.001 |
| by area sampled | 4452.6 | 0.581 | Mean distance to nearest town | -0.03 | 0.00 | -31.36 | <0.001 |
|  |  |  | Mean VRM | -224.10 | 15.61 | -14.36 | <0.001 |
|  |  |  | intercept | 3.30 | 0.06 | 57.60 | <0.001 |
| by area sampled | 4533.7 | 0.563 | Mean distance to nearest town | -0.03 | 0.00 | -17.88 | <0.001 |
|  |  |  | Distance to nearest trail | -0.13 | 0.01 | -11.31 | <0.001 |
|  |  |  | intercept | 3.63 | 0.05 | 72.68 | <0.001 |
| by area sampled | 4550.4 | 0.559 | Mean distance to nearest town | -0.04 | 0.00 | -40.83 | <0.001 |
|  |  |  | Distance to nearest 6th-order stream | 0.03 | 0.00 | 10.43 | <0.001 |
|  |  |  | intercept | 3.04 | 0.05 | 63.61 | <0.001 |
| by area sampled | 4558.9 | 0.557 | Distance to nearest trail | -0.31 | 0.01 | -40.81 | <0.001 |
|  |  |  | TRI | -0.04 | 0.00 | -16.98 | <0.001 |
|  |  |  | intercept | 3.93 | 0.06 | 66.67 | <0.001 |
| by area sampled | 4608.8 | 0.546 | Mean distance to nearest town | -0.04 | 0.00 | -46.33 | <0.001 |
|  |  |  | TRI | -0.02 | 0.00 | -7.07 | <0.001 |
|  |  |  | intercept | 3.25 | 0.09 | 37.32 | <0.001 |
| by area sampled | 4627.4 | 0.542 | Mean distance to nearest town | -0.04 | 0.00 | -37.86 | <0.001 |
|  |  |  | Elevation | 0.00 | 0.00 | 5.80 | <0.001 |
|  |  |  | intercept | 3.31 | 0.09 | 35.06 | <0.001 |
| by area sampled | 4641.3 | 0.539 | Mean distance to nearest town | -0.04 | 0.00 | -43.40 | <0.001 |
|  |  |  | Mean TRI | 0.03 | 0.01 | 4.43 | <0.001 |
|  |  |  | intercept | 2.83 | 0.20 | 14.12 | <0.001 |
| by area sampled | 4642.3 | 0.539 | Mean distance to nearest town | -0.04 | 0.00 | -45.67 | <0.001 |
|  |  |  | Mean annual precipitation | 0.00 | 0.00 | 4.31 | <0.001 |
|  |  |  | intercept | 3.42 | 0.08 | 42.43 | <0.001 |
| by area sampled | 4645.5 | 0.538 | Mean distance to nearest town | -0.04 | 0.00 | -33.47 | <0.001 |
|  |  |  | VRM | 12.90 | 3.26 | 3.96 | <0.001 |
|  |  |  | intercept | 3.69 | 0.05 | 79.06 | <0.001 |
| by area sampled | 4655.8 | 0.536 | Mean distance to nearest town | -0.04 | 0.00 | -43.72 | <0.001 |
|  |  |  | Distance to nearest 5th-order stream | -0.01 | 0.01 | -2.24 | 0.025 |
|  |  |  | intercept | 3.67 | 0.05 | 78.96 | <0.001 |
| by area sampled | 4658.9 | 0.535 | Mean distance to nearest town | -0.04 | 0.00 | -45.53 | <0.001 |
|  |  |  | intercept | -0.40 | 0.20 | -2.04 | 0.042 |
| by area sampled | 4683.5 | 0.530 | Distance to nearest trail | -0.32 | 0.01 | -37.56 | <0.001 |
|  |  |  | Mean annual precipitation | 0.00 | 0.00 | 13.93 | <0.001 |
|  |  |  | intercept | 3.63 | 0.10 | 37.48 | <0.001 |
| by area sampled | 4683.7 | 0.529 | Distance to nearest trail | -0.30 | 0.01 | -40.47 | <0.001 |
|  |  |  | Mean TRI | -0.09 | 0.01 | -13.82 | <0.001 |
|  |  |  | intercept | 0.69 | 0.11 | 6.50 | <0.001 |
| by area sampled | 4703.5 | 0.525 | Elevation | 0.00 | 0.00 | -10.34 | <0.001 |
|  |  |  | Mean wind speed | 0.38 | 0.01 | 44.33 | <0.001 |
|  |  |  | intercept | -2.29 | 0.23 | -10.10 | <0.001 |
| by area sampled | 4730.8 | 0.519 | Mean annual precipitation | 0.00 | 0.00 | 9.03 | <0.001 |
|  |  |  | Mean wind speed | 0.43 | 0.01 | 44.79 | <0.001 |
|  |  |  | intercept | 1.89 | 0.05 | 41.91 | <0.001 |
| by area sampled | 4765.1 | 0.511 | Distance to nearest trail | -0.24 | 0.01 | -28.50 | <0.001 |
|  |  |  | VRM | 33.92 | 3.06 | 11.10 | <0.001 |
|  |  |  | intercept | 2.58 | 0.03 | 75.57 | <0.001 |
| by area sampled | 4768.5 | 0.511 | Distance to nearest trail | -0.29 | 0.01 | -39.67 | <0.001 |
|  |  |  | Distance to nearest 6th-order stream | -0.03 | 0.00 | -10.38 | <0.001 |
|  |  |  | intercept | -0.42 | 0.05 | -8.07 | <0.001 |
| by area sampled | 4787.5 | 0.506 | Distance to nearest 6th-order stream | 0.02 | 0.00 | 5.06 | <0.001 |
|  |  |  | Mean wind speed | 0.40 | 0.01 | 45.54 | <0.001 |
|  |  |  | intercept | -0.47 | 0.06 | -8.15 | <0.001 |
| by area sampled | 4788.5 | 0.506 | Distance to nearest 5th-order stream | 0.03 | 0.01 | 5.01 | <0.001 |
|  |  |  | Mean wind speed | 0.41 | 0.01 | 44.97 | <0.001 |
|  |  |  | intercept | -0.45 | 0.06 | -7.57 | <0.001 |
| by area sampled | 4796.4 | 0.504 | TRI | 0.01 | 0.00 | 4.05 | <0.001 |
|  |  |  | Mean wind speed | 0.40 | 0.01 | 45.92 | <0.001 |
|  |  |  | intercept | -0.34 | 0.05 | -6.94 | <0.001 |
| by area sampled | 4807.3 | 0.502 | VRM | -7.65 | 3.35 | -2.29 | 0.022 |
|  |  |  | Mean wind speed | 0.42 | 0.01 | 32.18 | <0.001 |
|  |  |  | intercept | -0.48 | 0.11 | -4.38 | <0.001 |
| by area sampled | 4809.4 | 0.502 | Mean TRI | 0.01 | 0.01 | 1.77 | 0.077 |
|  |  |  | Mean wind speed | 0.40 | 0.01 | 45.64 | <0.001 |
|  |  |  | intercept | -0.30 | 0.05 | -6.60 | <0.001 |
| by area sampled | 4810.5 | 0.501 | Mean wind speed | 0.40 | 0.01 | 45.78 | <0.001 |
|  |  |  | intercept | 3.02 | 0.09 | 32.21 | <0.001 |
| by area sampled | 4819.6 | 0.499 | Distance to nearest trail | -0.26 | 0.01 | -35.15 | <0.001 |
|  |  |  | Elevation | 0.00 | 0.00 | -7.80 | <0.001 |
|  |  |  | intercept | 2.30 | 0.02 | 98.08 | <0.001 |
| by area sampled | 4879.2 | 0.486 | Distance to nearest trail | -0.28 | 0.01 | -37.35 | <0.001 |
|  |  |  | intercept | 5.01 | 0.11 | 47.20 | <0.001 |
| by area sampled | 5167.9 | 0.422 | Mean VRM | -539.70 | 13.45 | -40.14 | <0.001 |
|  |  |  | Elevation | 0.00 | 0.00 | -18.21 | <0.001 |
|  |  |  | intercept | 7.68 | 0.27 | 28.80 | <0.001 |
| by area sampled | 5190.7 | 0.417 | Mean annual precipitation | 0.00 | 0.00 | -17.19 | <0.001 |
|  |  |  | Mean VRM | -608.90 | 14.94 | -40.76 | <0.001 |
|  |  |  | intercept | 1.60 | 0.10 | 15.75 | <0.001 |
| by area sampled | 5213.8 | 0.412 | Mean TRI | 0.14 | 0.01 | 16.96 | <0.001 |
|  |  |  | Mean VRM | -557.80 | 12.92 | -43.18 | <0.001 |
|  |  |  | intercept | 2.96 | 0.05 | 63.48 | <0.001 |
| by area sampled | 5327.2 | 0.386 | TRI | 0.03 | 0.00 | 13.54 | <0.001 |
|  |  |  | Mean VRM | -556.00 | 13.58 | -40.96 | <0.001 |
|  |  |  | intercept | 3.52 | 0.05 | 69.40 | <0.001 |
| by area sampled | 5332.3 | 0.385 | Distance to nearest 5th-order stream | -0.08 | 0.01 | -12.06 | <0.001 |
|  |  |  | Mean VRM | -512.90 | 12.48 | -41.10 | <0.001 |
|  |  |  | intercept | 3.07 | 0.05 | 62.09 | <0.001 |
| by area sampled | 5480.4 | 0.352 | Distance to nearest 6th-order stream | 0.01 | 0.00 | 4.71 | <0.001 |
|  |  |  | Mean VRM | -495.40 | 12.63 | -39.23 | <0.001 |
|  |  |  | intercept | 3.18 | 0.04 | 72.78 | <0.001 |
| by area sampled | 5500.1 | 0.347 | Mean VRM | -495.63 | 12.58 | -39.39 | <0.001 |
|  |  |  | intercept | -3.50 | 0.26 | -13.26 | <0.001 |
| by area sampled | 5584.9 | 0.329 | Mean annual precipitation | 0.00 | 0.00 | 16.32 | <0.001 |
|  |  |  | VRM | 108.40 | 2.77 | 39.18 | <0.001 |
|  |  |  | intercept | 1.03 | 0.04 | 25.72 | <0.001 |
| by area sampled | 5773.5 | 0.287 | TRI | -0.02 | 0.00 | -9.07 | <0.001 |
|  |  |  | VRM | 85.27 | 2.19 | 38.97 | <0.001 |
|  |  |  | intercept | 1.36 | 0.10 | 14.09 | <0.001 |
| by area sampled | 5815.5 | 0.278 | VRM | 85.83 | 2.26 | 37.90 | <0.001 |
|  |  |  | Mean TRI | -0.05 | 0.01 | -6.65 | <0.001 |
|  |  |  | intercept | 1.36 | 0.11 | 12.70 | <0.001 |
| by area sampled | 5824.5 | 0.276 | VRM | 78.83 | 2.34 | 33.75 | <0.001 |
|  |  |  | Elevation | 0.00 | 0.00 | -6.00 | <0.001 |
|  |  |  | intercept | 0.74 | 0.03 | 27.52 | <0.001 |
| by area sampled | 5859.2 | 0.268 | VRM | 82.30 | 2.23 | 36.85 | <0.001 |
|  |  |  | intercept | 3.29 | 0.11 | 30.45 | <0.001 |
| by area sampled | 6643.1 | 0.094 | Distance to nearest 6th-order stream | 0.04 | 0.00 | 14.96 | <0.001 |
|  |  |  | Elevation | 0.00 | 0.00 | -19.41 | <0.001 |
|  |  |  | intercept | 2.54 | 0.12 | 21.11 | <0.001 |
| by area sampled | 6767.4 | 0.066 | Mean TRI | 0.06 | 0.01 | 8.60 | <0.001 |
|  |  |  | Elevation | 0.00 | 0.00 | -16.63 | <0.001 |
|  |  |  | intercept | 3.90 | 0.24 | 16.50 | <0.001 |
| by area sampled | 6820.9 | 0.054 | Mean annual precipitation | 0.00 | 0.00 | -4.33 | <0.001 |
|  |  |  | Elevation | 0.00 | 0.00 | -14.90 | <0.001 |
|  |  |  | intercept | 2.96 | 0.11 | 27.28 | <0.001 |
| by area sampled | 6829.4 | 0.052 | Distance to nearest 5th-order stream | -0.02 | 0.01 | -3.13 | 0.002 |
|  |  |  | Elevation | 0.00 | 0.00 | -13.36 | <0.001 |
|  |  |  | intercept | 2.99 | 0.11 | 27.69 | <0.001 |
| by area sampled | 6837.6 | 0.050 | Elevation | 0.00 | 0.00 | -14.81 | <0.001 |
|  |  |  | intercept | 1.39 | 0.03 | 44.00 | <0.001 |
| by area sampled | 6941.0 | 0.027 | Distance to nearest 5th-order stream | -0.05 | 0.01 | -8.37 | <0.001 |
|  |  |  | Distance to nearest 6th-order stream | 0.02 | 0.00 | 8.56 | <0.001 |
|  |  |  | intercept | 1.35 | 0.03 | 40.15 | <0.001 |
| by area sampled | 6986.8 | 0.017 | Distance to nearest 6th-order stream | 0.03 | 0.00 | 8.78 | <0.001 |
|  |  |  | TRI | -0.01 | 0.00 | -5.47 | <0.001 |
|  |  |  | intercept | 1.31 | 0.09 | 14.20 | <0.001 |
| by area sampled | 7005.1 | 0.013 | Distance to nearest 5th-order stream | -0.04 | 0.01 | -6.78 | <0.001 |
|  |  |  | Mean TRI | 0.02 | 0.01 | 2.46 | 0.014 |
|  |  |  | intercept | 1.58 | 0.04 | 39.50 | <0.001 |
| by area sampled | 7008.1 | 0.012 | Distance to nearest 5th-order stream | -0.05 | 0.01 | -7.09 | <0.001 |
|  |  |  | TRI | 0.00 | 0.00 | -1.72 | 0.086 |
|  |  |  | intercept | 1.53 | 0.03 | 59.49 | <0.001 |
| by area sampled | 7009.1 | 0.012 | Distance to nearest 5th-order stream | -0.05 | 0.01 | -6.98 | <0.001 |
|  |  |  | intercept | 1.24 | 0.03 | 46.10 | <0.001 |
| by area sampled | 7015.7 | 0.010 | Distance to nearest 6th-order stream | 0.02 | 0.00 | 6.89 | <0.001 |
|  |  |  | intercept | 0.79 | 0.11 | 7.43 | <0.001 |
| by area sampled | 7025.6 | 0.008 | TRI | -0.02 | 0.00 | -5.33 | <0.001 |
|  |  |  | Mean TRI | 0.06 | 0.01 | 6.11 | <0.001 |
|  |  |  | intercept | 2.67 | 0.24 | 10.98 | <0.001 |
| by area sampled | 7034.8 | 0.006 | Mean annual precipitation | 0.00 | 0.00 | -5.23 | <0.001 |
|  |  |  | TRI | -0.01 | 0.00 | -3.44 | <0.001 |
|  |  |  | intercept | 2.18 | 0.19 | 11.20 | <0.001 |
| by area sampled | 7045.0 | 0.004 | Mean annual precipitation | 0.00 | 0.00 | -4.10 | <0.001 |
|  |  |  | intercept | 1.12 | 0.09 | 12.66 | <0.001 |
| by area sampled | 7052.7 | 0.002 | Mean TRI | 0.02 | 0.01 | 3.02 | 0.003 |
|  |  |  | intercept | -1.87 | 0.42 | -4.46 | <0.001 |
| spatial weights | 479.8 | 0.738 | Distance to nearest trail | -0.13 | 0.02 | -6.66 | <0.001 |
|  |  |  | Mean annual temperature | 0.44 | 0.04 | 10.30 | <0.001 |
|  |  |  | intercept | -1.75 | 0.49 | -3.56 | <0.001 |
| spatial weights | 505.1 | 0.681 | Mean distance to nearest town | -0.01 | 0.00 | -5.25 | <0.001 |
|  |  |  | Mean annual temperature | 0.46 | 0.04 | 10.57 | <0.001 |
|  |  |  | intercept | -3.11 | 0.36 | -8.63 | <0.001 |
| spatial weights | 513.7 | 0.661 | Mean annual temperature | 0.47 | 0.04 | 10.87 | <0.001 |
|  |  |  | Mean wind speed | 0.12 | 0.03 | 4.33 | <0.001 |
|  |  |  | intercept | -3.04 | 0.38 | -8.08 | <0.001 |
| spatial weights | 518.2 | 0.651 | Mean annual temperature | 0.56 | 0.04 | 14.35 | <0.001 |
|  |  |  | TRI | -0.02 | 0.01 | -3.86 | <0.001 |
|  |  |  | intercept | -4.84 | 0.57 | -8.51 | <0.001 |
| spatial weights | 522.3 | 0.642 | Mean annual temperature | 0.61 | 0.04 | 14.73 | <0.001 |
|  |  |  | Mean TRI | 0.07 | 0.02 | 3.36 | <0.001 |
|  |  |  | intercept | 1.33 | 0.15 | 9.06 | <0.001 |
| spatial weights | 527.1 | 0.631 | Distance to nearest trail | -0.20 | 0.02 | -10.04 | <0.001 |
|  |  |  | Mean wind speed | 0.21 | 0.03 | 8.45 | <0.001 |
|  |  |  | intercept | -3.31 | 0.37 | -8.97 | <0.001 |
| spatial weights | 527.5 | 0.630 | Mean annual temperature | 0.54 | 0.04 | 12.96 | <0.001 |
|  |  |  | VRM | 19.16 | 7.74 | 2.48 | 0.013 |
|  |  |  | intercept | -4.52 | 0.60 | -7.52 | <0.001 |
| spatial weights | 528.4 | 0.628 | Mean annual temperature | 0.65 | 0.06 | 11.90 | <0.001 |
|  |  |  | Mean VRM | 81.05 | 35.66 | 2.27 | 0.023 |
|  |  |  | intercept | 3.01 | 0.10 | 30.38 | <0.001 |
| spatial weights | 530.8 | 0.623 | Distance to nearest trail | -0.21 | 0.02 | -11.24 | <0.001 |
|  |  |  | Mean VRM | -204.28 | 26.57 | -7.69 | <0.001 |
|  |  |  | intercept | -3.47 | 0.37 | -9.47 | <0.001 |
| spatial weights | 531.5 | 0.616 | Mean annual temperature | 0.57 | 0.04 | 14.56 | <0.001 |
|  |  |  | intercept | 3.58 | 0.15 | 24.68 | <0.001 |
| spatial weights | 538.5 | 0.605 | Mean distance to nearest town | -0.05 | 0.00 | -13.44 | <0.001 |
|  |  |  | Distance to nearest 6th-order stream | 0.07 | 0.01 | 9.06 | <0.001 |
|  |  |  | intercept | 3.01 | 0.11 | 27.81 | <0.001 |
| spatial weights | 550.5 | 0.578 | Distance to nearest trail | -0.26 | 0.02 | -13.12 | <0.001 |
|  |  |  | TRI | -0.04 | 0.01 | -6.89 | <0.001 |
|  |  |  | intercept | 3.07 | 0.13 | 23.36 | <0.001 |
| spatial weights | 568.6 | 0.537 | Mean distance to nearest town | -0.02 | 0.00 | -5.61 | <0.001 |
|  |  |  | Distance to nearest trail | -0.18 | 0.02 | -7.45 | <0.001 |
|  |  |  | intercept | 3.54 | 0.25 | 14.38 | <0.001 |
| spatial weights | 577.3 | 0.517 | Distance to nearest trail | -0.25 | 0.02 | -13.21 | <0.001 |
|  |  |  | Mean TRI | -0.09 | 0.02 | -4.78 | <0.001 |
|  |  |  | intercept | 2.15 | 0.11 | 19.39 | <0.001 |
| spatial weights | 595.4 | 0.477 | Distance to nearest trail | -0.23 | 0.02 | -10.57 | <0.001 |
|  |  |  | VRM | 21.47 | 8.27 | 2.60 | 0.009 |
|  |  |  | intercept | 1.80 | 0.25 | 7.21 | <0.001 |
| spatial weights | 596.0 | 0.475 | Distance to nearest trail | -0.27 | 0.02 | -11.86 | <0.001 |
|  |  |  | Elevation | 0.00 | 0.00 | 2.46 | 0.014 |
|  |  |  | intercept | 2.17 | 0.26 | 8.27 | <0.001 |
| spatial weights | 597.3 | 0.472 | Mean distance to nearest town | -0.02 | 0.00 | -7.62 | <0.001 |
|  |  |  | Mean wind speed | 0.16 | 0.03 | 5.36 | <0.001 |
|  |  |  | intercept | 2.39 | 0.06 | 37.23 | <0.001 |
| spatial weights | 599.9 | 0.462 | Distance to nearest trail | -0.25 | 0.02 | -12.37 | <0.001 |
|  |  |  | intercept | 3.53 | 0.14 | 25.93 | <0.001 |
| spatial weights | 601.0 | 0.464 | Mean distance to nearest town | -0.02 | 0.00 | -9.70 | <0.001 |
|  |  |  | Mean VRM | -139.53 | 28.36 | -4.92 | <0.001 |
|  |  |  | intercept | 2.07 | 0.11 | 18.61 | <0.001 |
| spatial weights | 621.6 | 0.417 | VRM | 63.78 | 6.99 | 9.13 | <0.001 |
|  |  |  | Mean VRM | -265.35 | 28.08 | -9.45 | <0.001 |
|  |  |  | intercept | -0.17 | 0.16 | -1.08 | 0.278 |
| spatial weights | 622.8 | 0.415 | Distance to nearest 6th-order stream | 0.04 | 0.01 | 5.75 | <0.001 |
|  |  |  | Mean wind speed | 0.33 | 0.03 | 12.51 | <0.001 |
|  |  |  | intercept | 3.44 | 0.14 | 25.42 | <0.001 |
| spatial weights | 624.4 | 0.411 | Mean distance to nearest town | -0.03 | 0.00 | -12.06 | <0.001 |
|  |  |  | TRI | -0.01 | 0.01 | -1.81 | 0.070 |
|  |  |  | intercept | 4.23 | 0.53 | 8.02 | <0.001 |
| spatial weights | 624.9 | 0.410 | Mean distance to nearest town | -0.03 | 0.00 | -12.56 | <0.001 |
|  |  |  | Mean annual precipitation | 0.00 | 0.00 | -1.68 | 0.092 |
|  |  |  | intercept | 1.09 | 0.19 | 5.67 | <0.001 |
| spatial weights | 625.1 | 0.410 | Mean VRM | -140.15 | 27.42 | -5.11 | <0.001 |
|  |  |  | Mean wind speed | 0.24 | 0.03 | 8.38 | <0.001 |
|  |  |  | intercept | 3.37 | 0.13 | 26.16 | <0.001 |
| spatial weights | 625.7 | 0.404 | Mean distance to nearest town | -0.03 | 0.00 | -13.22 | <0.001 |
|  |  |  | intercept | 1.26 | 0.28 | 4.52 | <0.001 |
| spatial weights | 639.6 | 0.377 | Elevation | 0.00 | 0.00 | -3.80 | <0.001 |
|  |  |  | Mean wind speed | 0.29 | 0.02 | 11.89 | <0.001 |
|  |  |  | intercept | 0.67 | 0.27 | 2.46 | 0.014 |
| spatial weights | 643.9 | 0.367 | Mean TRI | 0.18 | 0.02 | 7.24 | <0.001 |
|  |  |  | Mean VRM | -387.15 | 31.63 | -12.24 | <0.001 |
|  |  |  | intercept | 0.59 | 0.16 | 3.82 | <0.001 |
| spatial weights | 645.2 | 0.364 | TRI | -0.01 | 0.00 | -2.98 | 0.003 |
|  |  |  | Mean wind speed | 0.29 | 0.03 | 11.27 | <0.001 |
|  |  |  | intercept | 0.05 | 0.16 | 0.31 | 0.758 |
| spatial weights | 647.8 | 0.358 | Distance to nearest 5th-order stream | 0.03 | 0.01 | 2.60 | 0.009 |
|  |  |  | Mean wind speed | 0.33 | 0.03 | 11.94 | <0.001 |
|  |  |  | intercept | 0.31 | 0.13 | 2.45 | 0.015 |
| spatial weights | 652.2 | 0.344 | Mean wind speed | 0.30 | 0.03 | 12.00 | <0.001 |
|  |  |  | intercept | 3.98 | 0.28 | 14.21 | <0.001 |
| spatial weights | 666.1 | 0.317 | Mean VRM | -280.80 | 29.08 | -9.65 | <0.001 |
|  |  |  | Elevation | 0.00 | 0.00 | -5.52 | <0.001 |
|  |  |  | intercept | 1.67 | 0.09 | 18.06 | <0.001 |
| spatial weights | 677.4 | 0.291 | TRI | -0.04 | 0.00 | -7.46 | <0.001 |
|  |  |  | VRM | 71.45 | 6.51 | 10.97 | <0.001 |
|  |  |  | intercept | 4.45 | 0.55 | 8.04 | <0.001 |
| spatial weights | 683.8 | 0.277 | Mean annual precipitation | 0.00 | 0.00 | -3.57 | <0.001 |
|  |  |  | Mean VRM | -254.10 | 27.57 | -9.22 | <0.001 |
|  |  |  | intercept | 2.64 | 0.11 | 24.61 | <0.001 |
| spatial weights | 689.1 | 0.265 | Distance to nearest 5th-order stream | -0.03 | 0.01 | -2.70 | 0.007 |
|  |  |  | Mean VRM | -249.55 | 26.37 | -9.46 | <0.001 |
|  |  |  | intercept | 2.50 | 0.10 | 26.19 | <0.001 |
| spatial weights | 694.7 | 0.248 | Mean VRM | -248.31 | 26.67 | -9.31 | <0.001 |
|  |  |  | intercept | 0.84 | 0.10 | 8.47 | <0.001 |
| spatial weights | 717.4 | 0.201 | Distance to nearest 6th-order stream | 0.03 | 0.01 | 4.70 | <0.001 |
|  |  |  | VRM | 66.72 | 7.24 | 9.22 | <0.001 |
|  |  |  | intercept | 1.83 | 0.23 | 8.11 | <0.001 |
| spatial weights | 727.5 | 0.178 | VRM | 63.62 | 6.95 | 9.15 | <0.001 |
|  |  |  | Mean TRI | -0.05 | 0.02 | -3.19 | 0.001 |
|  |  |  | intercept | 0.97 | 0.12 | 8.44 | <0.001 |
| spatial weights | 734.8 | 0.162 | Distance to nearest 5th-order stream | 0.03 | 0.01 | 1.88 | 0.060 |
|  |  |  | VRM | 68.46 | 7.95 | 8.61 | <0.001 |
|  |  |  | intercept | 1.14 | 0.07 | 15.63 | <0.001 |
| spatial weights | 736.2 | 0.154 | VRM | 61.43 | 7.05 | 8.71 | <0.001 |
|  |  |  | intercept | 5.52 | 0.60 | 9.17 | <0.001 |
| spatial weights | 743.7 | 0.142 | Mean annual precipitation | 0.00 | 0.00 | -5.92 | <0.001 |
|  |  |  | TRI | -0.03 | 0.01 | -6.58 | <0.001 |
|  |  |  | intercept | 3.51 | 0.29 | 12.05 | <0.001 |
| spatial weights | 749.9 | 0.128 | TRI | -0.03 | 0.00 | -6.13 | <0.001 |
|  |  |  | Elevation | 0.00 | 0.00 | -5.39 | <0.001 |
|  |  |  | intercept | 1.78 | 0.09 | 20.27 | <0.001 |
| spatial weights | 755.0 | 0.116 | Distance to nearest 6th-order stream | 0.03 | 0.01 | 5.01 | <0.001 |
|  |  |  | TRI | -0.03 | 0.00 | -6.05 | <0.001 |
|  |  |  | intercept | 2.29 | 0.11 | 20.66 | <0.001 |
| spatial weights | 762.9 | 0.098 | Distance to nearest 5th-order stream | -0.05 | 0.01 | -3.87 | <0.001 |
|  |  |  | TRI | -0.03 | 0.00 | -6.08 | <0.001 |
|  |  |  | intercept | 1.44 | 0.24 | 5.91 | <0.001 |
| spatial weights | 773.5 | 0.075 | TRI | -0.03 | 0.01 | -5.17 | <0.001 |
|  |  |  | Mean TRI | 0.05 | 0.02 | 2.33 | 0.020 |
|  |  |  | intercept | 2.53 | 0.28 | 9.14 | <0.001 |
| spatial weights | 776.7 | 0.067 | Distance to nearest 6th-order stream | 0.02 | 0.01 | 3.83 | <0.001 |
|  |  |  | Elevation | 0.00 | 0.00 | -4.11 | <0.001 |
|  |  |  | intercept | 1.97 | 0.08 | 25.08 | <0.001 |
| spatial weights | 776.9 | 0.062 | TRI | -0.02 | 0.00 | -5.16 | <0.001 |
|  |  |  | intercept | 3.92 | 0.55 | 7.07 | <0.001 |
| spatial weights | 784.0 | 0.051 | Mean annual precipitation | 0.00 | 0.00 | -2.59 | 0.010 |
|  |  |  | Elevation | 0.00 | 0.00 | -2.71 | 0.007 |
|  |  |  | intercept | 4.46 | 0.62 | 7.22 | <0.001 |
| spatial weights | 784.3 | 0.050 | Mean annual precipitation | 0.00 | 0.00 | -4.19 | <0.001 |
|  |  |  | Mean TRI | -0.04 | 0.02 | -2.62 | 0.009 |
|  |  |  | intercept | 3.08 | 0.58 | 5.27 | <0.001 |
| spatial weights | 785.6 | 0.047 | Distance to nearest 6th-order stream | 0.01 | 0.01 | 2.47 | 0.014 |
|  |  |  | Mean annual precipitation | 0.00 | 0.00 | -2.86 | 0.004 |
|  |  |  | intercept | 3.07 | 0.33 | 9.23 | <0.001 |
| spatial weights | 786.6 | 0.045 | Mean TRI | -0.03 | 0.02 | -1.97 | 0.048 |
|  |  |  | Elevation | 0.00 | 0.00 | -3.90 | <0.001 |
|  |  |  | intercept | 1.54 | 0.08 | 18.52 | <0.001 |
| spatial weights | 787.2 | 0.044 | Distance to nearest 5th-order stream | -0.03 | 0.01 | -2.50 | 0.012 |
|  |  |  | Distance to nearest 6th-order stream | 0.02 | 0.01 | 3.97 | <0.001 |
|  |  |  | intercept | 2.68 | 0.27 | 9.87 | <0.001 |
| spatial weights | 788.6 | 0.036 | Elevation | 0.00 | 0.00 | -3.96 | <0.001 |
|  |  |  | intercept | 1.88 | 0.22 | 8.44 | <0.001 |
| spatial weights | 788.9 | 0.040 | Distance to nearest 6th-order stream | 0.02 | 0.01 | 3.72 | <0.001 |
|  |  |  | Mean TRI | -0.04 | 0.02 | -2.18 | 0.030 |
|  |  |  | intercept | 3.68 | 0.53 | 6.90 | <0.001 |
| spatial weights | 789.5 | 0.034 | Mean annual precipitation | 0.00 | 0.00 | -3.87 | <0.001 |
|  |  |  | intercept | 1.41 | 0.07 | 20.57 | <0.001 |
| spatial weights | 791.8 | 0.029 | Distance to nearest 6th-order stream | 0.02 | 0.01 | 3.66 | <0.001 |
|  |  |  | intercept | 2.20 | 0.22 | 9.93 | <0.001 |
| spatial weights | 796.9 | 0.022 | Distance to nearest 5th-order stream | -0.03 | 0.01 | -2.23 | 0.026 |
|  |  |  | Mean TRI | -0.04 | 0.02 | -2.25 | 0.025 |
|  |  |  | intercept | 2.04 | 0.21 | 9.67 | <0.001 |
| spatial weights | 800.0 | 0.010 | Mean TRI | -0.03 | 0.02 | -2.09 | 0.037 |
|  |  |  | intercept | 1.72 | 0.07 | 25.73 | <0.001 |
| spatial weights | 800.1 | 0.010 | Distance to nearest 5th-order stream | -0.03 | 0.01 | -2.06 | 0.039 |
